# Natural herbal extract roles and mechanisms in treating cerebral ischemia: A systematic review

**DOI:** 10.3389/fphar.2024.1424146

**Published:** 2024-08-02

**Authors:** Jiashuo Yang, Bo Yu, Jian Zheng

**Affiliations:** Department of Neurosurgery, Shengjing Hospital of China Medical University, Shenyang, China

**Keywords:** natural herbal extract, herbs, cerebral ischemia, blood-brain barrier, flavonoids

## Abstract

**Background:**

Stroke has been the focus of medical research due to its serious consequences and sequelae. Among the tens of millions of new stroke patients every year, cerebral ischemia patients account for the vast majority. While cerebral ischemia drug research and development is still ongoing, most drugs are terminated at preclinical stages due to their unacceptable toxic side effects. In recent years, natural herbs have received considerable attention in the pharmaceutical research and development field due to their low toxicity levels. Numerous studies have shown that natural herbs exert actions that cannot be ignored when treating cerebral ischemia.

**Methods:**

We reviewed and summarized the therapeutic effects and mechanisms of different natural herbal extracts on cerebral ischemia to promote their application in this field. We used keywords such as “natural herbal extract,” “herbal medicine,” “Chinese herbal medicine” and “cerebral ischemia” to comprehensively search PubMed, ScienceDirect, ScienceNet, CNKI, and Wanfang databases, after which we conducted a detailed screening and review strategy.

**Results:**

We included 120 high-quality studies up to 10 January 2024. Natural herbal extracts had significant roles in cerebral ischemia treatments *via* several molecular mechanisms, such as improving regional blood flow disorders, protecting the blood-brain barrier, and inhibiting neuronal apoptosis, oxidative stress and inflammatory responses.

**Conclusion:**

Natural herbal extracts are represented by low toxicity and high curative effects, and will become indispensable therapeutic options in the cerebral ischemia treatment field.

## Graphical Abstract

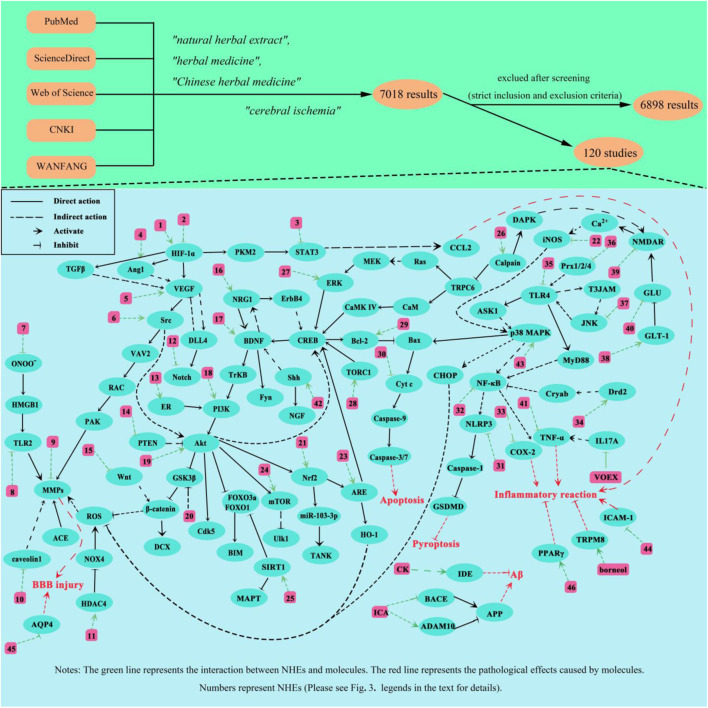

## 1 Introduction

Cerebral ischemia (CI) is a complex disease in clinical medicine. To put it simply, due to various reasons, blood in brain tissue cannot support normal metabolism and function, with subsequent symptoms collectively referred to as CI. Worldwide, morbidity and mortality rates due to CI are very high. The disease is characterized by several etiologies, changeable conditions, and serious consequences, which exert extremely heavy burdens on patients and their families. The National Institutes of Health Stroke Scale is commonly used to assess neurological damage in patients with clinical ischemia. Even if a patient avoids death, most will experience severe neurological dysfunction. Currently, the early treatment of patients with CI mainly occurs *via* the rapid restoration of cerebral blood flow perfusion, however, this restoration increases oxidative stress and inflammatory responses in ischemic tissue, leading to reperfusion injury. One factor that determines the severity of a patient’s condition is ischemia duration, which is a very important determinant when selecting treatment options for patients with acute cerebral ischemia (ACI) (Powers, 2020). Another problem that cannot be ignored is that the recombinant tissue plasminogen activator (rt-PA) drug is currently approved by the U.S. Food and Drug Administration for stroke patients, but its treatment window is very narrow and it has very serious side effects (e.g., cerebral hemorrhaging) (Dhamija and Donnan, 2007). Thus, a lack of drugs is an urgent issue for CI treatment. To remedy this, promoting low-toxicity and high-efficiency drug research and development can alleviate CI patient suffering. Our work is based on this purpose and motivation.

Herbal medicines are gifts from nature, and have helped humans solve medical problems that have plagued humankind for centuries. For example, artemisinin extracted from *Artemisia annua* L. helps alleviate malaria (Talman et al., 2019). Methanol extracts from *Allium turcicum* Özhatay and Cowley exert significant anticancer, antioxidant, and antimicrobial activities (łpek et al., 2024). These natural herbal extracts (NHEs) have active roles in many different fields, for example, a *Chenopodium quinoa* Willd. seed extract restores photosystem II damage caused by toxic metal salts (Ganieva et al., 2023). Also, *Pistacia atlantica* Desf. extracts effectively inhibit *Fusarium oxysporum f*. *sp. albedinis* to rescue infected date palms (Fatiha et al., 2023). NHEs have unique structures and properties, and are roughly divided into alkaloids, flavonoids, polysaccharides, glycosides, organic acids, and volatile oils.

### 1.1 Alkaloids

Alkaloids are nitrogen-containing organic compounds mainly found in plants, which have similar chemical properties to alkalis. One common property is that they all contain nitrogen as part of their chemical structure, but not all organic compounds containing nitrogen are alkaloids. Thanks to natural herb research and exploration, nearly 10,000 alkaloids have been discovered and collected. Alkaloids are subdivided into more than 60 types, for example, *Leonurus japonicus* Houtt. total alkaloid (LHA) is an organic amine in alkaloids. LHA and kukoamine A (KuA) are common alkaloids. LHA helps protect the blood-brain barrier (BBB) and inhibits inflammatory reactions and apoptosis in ischemia-reperfusion (IR) injury (Zhang Q.-Y. et al., 2017; Li Y. et al., 2021). KuA impacts CI injury by alleviating brain edema and inhibiting oxidative stress and apoptosis (Liu et al., 2017).

### 1.2 Flavonoids

Flavonoids are one of the most widespread organic compounds in nature; they exist in almost all green plants, especially higher plants. In the selected studies in this review, many have investigated flavonoid NHEs, such as emodin, scutellarin, and icariin (ICA). From our research, emodin enhances cell viability and inhibits oxidative stress radicalization to alleviate IR injury (Wang et al., 2007; Leung et al., 2020); scutellarin improves neurological dysfunction in rats during IR injury by inhibiting apoptosis, focal death, and necrosis (Wang C. et al., 2023); and ICA inhibits apoptosis and protects neuronal dendrites during chronic cerebral ischemia (CCI) to improve cognitive impairment (Li W.-X. et al., 2015).

### 1.3 Polysaccharides

Polysaccharides are macromolecules composed of at least 10 monosaccharides, and have important roles maintaining normal life activities. Polysaccharides are divided into plant, animal, and fungal polysaccharides according to extraction sources. Plant polysaccharides include ganoderma polysaccharides, lentinan, ginseng polysaccharides, and other polysaccharides beneficial to humans. Previous studies have reported that some polysaccharides may have anti-tumor effects (Zhang et al., 2021).

### 1.4 Glycosides

Glycosides have high medicinal value, which not only enhance immunity and antiviral effects, but also inhibit oxidative stress and enhance metabolic function in cells. Astragaloside IV (ASIV), ginsenoside, and notoginsenoside are well-known representative glycosides, especially ASIV. As an *Astragalus membranaceus* (Fisch.) Bunge extract, ASIV inhibits inflammatory reactions, promotes neurogenesis and angiogenesis, promotes neurotrophic factor expression, protects the BBB, and significantly improves neurological dysfunction caused by IR injury (Li et al., 2013; Li L. et al., 2021; Li S. et al., 2021; Shi et al., 2021). Ginsenoside has protective roles in injury caused by ACI, CCI, and IR. It not only inhibits apoptosis, increases angiogenesis, and improves local blood flow disorders, but also protects the BBB and improves cognitive dysfunction caused by CI (Zhou et al., 2014; Yang et al., 2016; Wang S. et al., 2017; Wan et al., 2017; Zhang et al., 2019; Zhang C. et al., 2020). Notoginsenoside resists injury caused by ACI and IR by alleviating brain edema, enhancing cell viability, protecting the BBB, and inhibiting apoptosis (Tu et al., 2018; Liu B. et al., 2021; Gao et al., 2022; Liu et al., 2022).

### 1.5 Organic acids

Organic acids are widely found in leaves, roots, and especially plant fruits. They have acidic properties, and are widely found in *Lonicera japonica* Thunb., *Schisandra chinensis* (Turcz.) Baill., *Prunus mume* Siebold & Zucc., *Rubus idaeus* L., and other herbs. Representative organic acid compounds from NHEs include salvianolic acid A (SAA) and betulinic acid (BA). In an ACI model, SAA reduces the incidence of cerebral hemorrhaging, protects the BBB, relieves vascular endothelial dysfunction, promotes neural function recovery, and induces neural progenitor cell proliferation. SAA also alleviates ischemic brain edema, inhibits inflammatory reactions, relieves oxidative stress, inhibits apoptosis, and improves long-term learning and memory defects (Jiang et al., 2011; Chien et al., 2016; Song et al., 2019; Liu C. et al., 2021; Ling et al., 2021; Yang Y. et al., 2022). BA also inhibits neuronal autophagy against IR injury (Zhao et al., 2021).

### 1.6 Volatile oils

Volatile oils mainly come from aromatic traditional Chinese medicine, with many fragrant plants more or less containing these compounds. The group includes terpenoids and aromatic compounds and also their oxygen-containing derivatives such as alcohols, aldehydes, ketones, phenols, ethers, and lipids. Additionally, the group includes some nitrogen- and sulfur-containing compounds. The most common NHEs are ginkgolide and ligustilide (LIG). Seven ginkgolide species have been found: A, B, C, M, J, K, and L. Ginkgolide B (GB) exerts the greatest effects, so many pharmacological studies have focused on this compound. GB protects the BBB and improves mitochondrial respiratory function against ACI injury (Li et al., 2007). LIG alleviates neurological deficits and inhibits apoptosis, astrocyte activation and proliferation, and oxidative stress responses in a CCI model (Feng et al., 2012; Peng D. et al., 2022).

## 2 Search strategy

**FIGURE 1:**
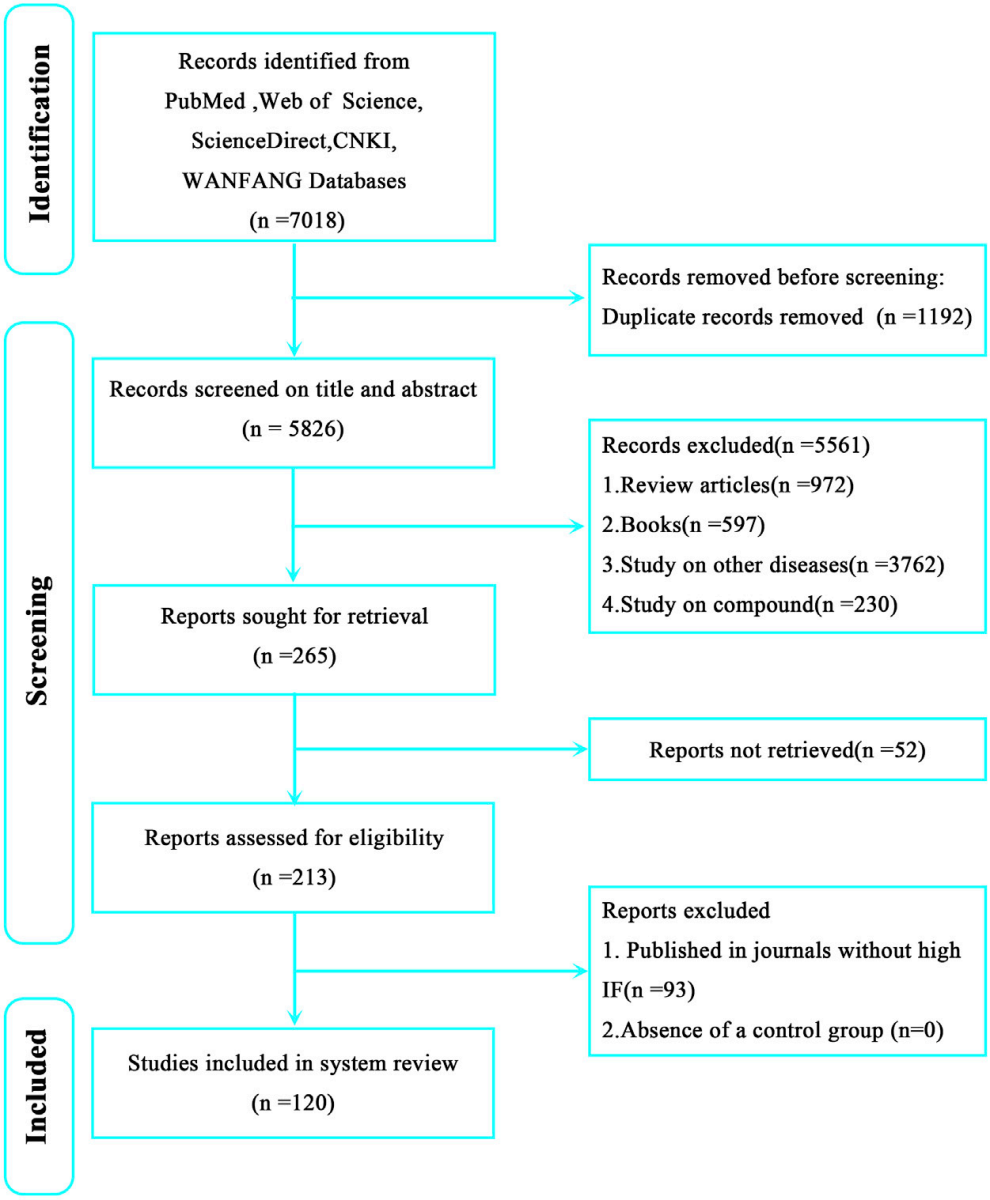
Preferred Reporting Items for Systematic Review and Meta-analysis (PRISMA) flow chart showing literature retrieval and screening in this review.

To comprehensively and systematically conduct literature retrieval and data extraction, we preliminarily searched and screened all studies from PubMed, ScienceDirect, Web of Science, CNKI, and WANFANG databases before 10 January 2024 in strict accordance with Preferred Reporting Items for Systematic Review and Meta-analysis (PRISMA) guidelines. The retrieval keywords are as follows: 1) natural herbal extracts, 2) Chinese herbal medicines, 3) herbal medicines, and 4) cerebral ischemia. Constructed retrieval expressions are (natural herbal extracts OR Chinese herbal medicines OR herbal medicines) AND cerebral ischemia. After retrieving pertinent studies (*n* = 7,018), we preliminarily screened (using titles, keywords, and abstracts) and comprehensively reviewed these studies. Finally, 120 studies were selected for review (Figure 1).

### 2.1 Inclusion and exclusion criteria

In view of the therapeutic effects and mechanisms of NHEs toward CI, and the large number of studies, we formulated the following inclusion and exclusion criteria. Inclusion criteria; 1) A cerebral ischemia model and 2) a control group are included; 3) At least one experimental group used NHEs as an intervention; and 4) Research data are published in high impact journals. Exclusion criteria; 1) Reviews or books; and 2) Studies on other diseases and compounds.

### 2.2 Data extraction and treatment evaluation

The authors independently extracted and summarized information from selected studies, solved any issues *via* discussion, and finally summarized the information, including; 1) NHEs; 2) The source and voucher numbers of the herbal medicine; 3) Extraction methods 4) Extraction parts and solvents; 5) Toxicity and side effects; 6) Related diseases in the study; 7) Establishing *in vitro* or *in vivo* models; 8) Animal or cell models; 9) Dose and time of treatment; 10) Main biological effects; 11) Mechanism of action; 12) Year of publication and first author; and 13) Positive controls.

### 2.3 Risk of bias

After discussions, the authors referred to the previous literature (Dong et al., 2023) to establish an evaluation scale of bias risk for selected studies. The following questions were posed; 1) Had the study passed peer review; 2) Were randomness principles followed when grouping models; 3) Were blinding methods applied during drug interventions and data collection; 4) Were sample sizes statistically calculated before model establishment; 5) Were animal welfare laws and regulations strictly observed in the research process; and 6) Were potential conflicts of interest between authors declared?

## 3 Results

From our strategy, 7,018 studies were retrieved, including 2032 Pubmed results, 1,242 Web of Science results, 3,578 ScienceDirect results, 124 CNKI results, and 42 WANFANG results. After preliminary screening and applying inclusion and exclusion, 120 studies were finally selected (Figure 1). Among these, 21 were related to ACI, 28 to CCI, and 71 to IR. In order to avoid disease subtype differences which may have affected our study results, we separately summarized the therapeutic effects of NHEs for ACI, CCI, and IR.

### 3.1 ACI

In the 21 ACI-related studies were 19 *in vivo* and nine *in vitro* studies, from which we summarized the characteristics of each study (Table 1). To ensure reliability, we summarized *in vivo* and *in vitro* study characteristics, separately. In the 19 *in vivo* studies, NHEs mitigated ACI-induced damage *via* different modes of action (Table 2). In nine *in vitro* studies, damaged cell models achieved varying degrees of remission after a NHE intervention (Table 3).

**TABLE 1:** Summary of ACI study characteristics.

*In vivo*		Quantity	*In vitro*		Quantity
Model			Model		
	MCAO	14		OGD	7
	PT	3		Model of cell injury induced by COCl_2_	1
	autologous thrombotic stroke model	2	Cell types		
Species				PC12	2
	SD rats	15		N2A	1
	ICR mice	2		bEnd.3	1
	Wistar rats	1		HBMEC	1
	Tree shrews	1		Primary neurons	2
	C57BL/6 mice	1		Primary microglia	1
Drug effect				Hippocampal slices	1
	Reduce neurological deficit	15	Drug effect		
	Reduce cerebral edema	4		Enhance cell viability	7
	Inhibit oxidative stress response	4		Improve mitochondrial dysfunction	1
	Reduce infarct size	11		Protect the blood-brain barrier	1
	Inhibition inflammatory response	3		Inhibit calcium inflow	1
	Reduce cerebral thrombosis	1		Inhibit oxidative stress response	1
	Protect the blood-brain barrier	3		Inhibit apoptosis	2
	Ameliorate mitochondrial dysfunction	3		Inhibit inflammatory response	2
	Improve regional cerebral blood flow disturbance	5		Alleviate glutamate hyperexcitation injury	1
	Promote angiogenesis	2			
	Inhibit astrocyte activation and proliferation	1			

**TABLE 2:** NHE therapeutic effects and mechanisms in *in vivo* ACI models.

Author(Year)	Extracts	Model	Species	Interventions	Positive control	Biological effects (experimental protocol)	Mechanism	Regulation
Wang et al. (2022)	catalpol	MCAO	SD rats	catalpol(10 mg/kg) for 7 d	NA	Reduce neurological deficit (mNSS) Alleviate ischemic brain edema (water content calculation) Inhibit oxidative stress response (MDA assay) Reduce infarct size (TTC)	Nrf2/HO-1 path Bax/Bcl-2 path	Upregulated(Nrf2/HO-1 path) Downregulated(Bax/Bcl-2 path)
Zhao et al. (2017)	MO	autologous thrombotic stroke model	Wistar rats	MO(100/250/500 mg/kg)for 3 d	NA	Reduce neurological deficit (neurological deficit scores) Reduce infarct size (TTC) Reduce cerebral thrombosis (radioimmunoassay) Inhibit oxidative stress and inflammatory response (Westernblot)	6-keto-PGF1α/TXB2 Bax/Bcl-2 path	Downregulated
Li et al. (2007)	GB	PT	Tree shrews	GB(5 mg/kg)for 6 h	NA	Protect the blood-brain barrier (EB test) Improve the mitochondrial respiration (determining the oxygen consumption in an airtight chamber)	PAFR	Downregulated
Fei et al. (2017)	SCED	MCAO	SD rats	SCED(3.75/7.5/15 mg/kg) for 3 d	Ginaton(15 mg/kg)	Reduce neurological deficit (Longa) Improve regional cerebral blood flow disturbance (laser-Doppler) Alleviate ischemic brain edema (water content calculation) Reduce infarct size (TTC)	TXA2 PLC/PKC path	Downregulated
Liu et al. (2022)	NGR1	MCAO	SD rats	NGR1(20/40 mg/kg) for 24 h	Dl-3-n-Butylphthalide	Reduce neurological deficit (neurological deficit scores) Reduce infarct size (TTC) Accelerate energy metabolism (RT-qPCR)	Atp12a Atp6v1g3	Upregulated
Gao et al. (2022)	PNS	MCAO	ICR mice	PNS(50/100 mg/kg) for 3 d	minocycline	Reduce neurological deficit (Longa) Improve regional cerebral blood flow disturbance (Laser speckle imaging) Inhibit microglial activation and inflammatory response (Westernblot)	HIF-1α/PKM2/STAT3 path	Downregulated
Liu et al. (2021a)	NGR1	MCAO	SD rats	NGR1(10/20/40 mg/kg) for 12 h	Dl-3-n-Butylphthalide	Protect the blood-brain barrier (EB test)	NA	
Wang et al. (2017a)	GSRb1	PT	SD rats	GSRb1(25/50/100 mg/kg) for 1 d	nimodipine	Improve regional cerebral blood flow disturbance (laser-Doppler)	GLT-1 NMDAR Cyt-C	Upregulated(GLT-1) Downregulated(NMDAR and Cyt-C)
Liu et al. (2021b)	SAA	autologous thrombotic stroke model	SD rats	SAA(10 mg/kg) for 5 d	aspirin	Reduce neurological deficit (Longa) Protect the blood-brain barrier (EB test)	VEGFA/Src/VAV2/Rac/PAK/MMPs	Downregulated
Liu et al. (2010)	TSA	MCAO	SD rats	TSA(15/20 mg/kg) for 1 d	NA	Reduce neurological deficit (Longa) Alleviate ischemic brain edema (water content calculation)	TORC1/CREB/BDNF path	Upregulated
Yang et al. (2016)	GSRd	MCAO	SD rats	GSRd(10 mg/kg) for 7 d	NA	Reduce neurological deficit (Longa) Reduce infarct size (TTC) Mitigate mitochondrial DNA and nuclear DNA damage (real-time analysis of mutation frequency)	NEIL1/3	Upregulated
Ma et al. (2021)	L-borneol	MCAO	SD rats	L-borneol(50/100/200 mg/kg) for 3 d	nimodipine(12 mg/kg)	Reduce neurological deficit (Longa) Reduce infarct size (TTC) Promote angiogenesis (ELISA)	Ang1/VEGF/BDNF path	Upregulated
Zhang et al. (2023b)	SHPL-49	MCAO	SD rats	SHPL-49(2.5/5/7.5/10/15 mg/kg) for 5 d	Edaravone(7.5 mg/kg)	Improve regional cerebral blood flow disturbance (laser-Doppler) Reduce infarct size (TTC) Reduce neurological deficit (Bederson)	Bax/Bcl-2/Caspase-3 path	Downregulated
Li et al. (2012a)	Galangin	MCAO	SD rats	Galangin(25/50/100 mg/kg)	EGB761(4 mg/kg)	Improve mitochondrial viability (Measurement of Mitochondrial Viability) Improve regional cerebral blood flow disturbance (laser-Doppler) Inhibit oxidative stress response (ROS assay) Reduce infarct size (TTC)	Bax/Bcl-2/Caspase-3 path	Downregulated
Liu et al. (2017)	KuA	MCAO	SD rats	KuA(5/10/20 mg/kg) for 6 h	NA	Reduce neurological deficit (neurological deficit scores) Reduce cerebral edema (water content calculation) Inhibit oxidative stress response (MDA assay) Reduce infarct size (TTC)	Bax/Bcl-2/Caspase-3 path	Downregulated
Li et al. (2015a)	T-VA	MCAO	SD rats ICR mice	T-VA(30/60/120 mg/kg) for 10 d	NA	Reduce neurological deficit (neurological deficit scores) Promote vascular endothelial cell proliferation (immunohistochemical)	VEGF	Upregulated
Liu et al. (2019a)	KRGP	MCAO	C57BL/6 mice	KRGP(100 mg/kg) for 7 d	NA	Reduce neurological deficit (neurological deficit scores) Inhibit astrocyte activation and proliferation (immunofluorescence) Ameliorate abnormal glutamate metabolism (Westernblot)	Nrf2	Upregulated
Jiang et al. (2018a)	Celastro	MCAO	SD rats	celastro	NA	Reduce neurological deficit (neurological deficit scores) Reduce infarct size (TTC) Inhibit inflammatory response (immunofluorescence)	NA	
Zhang et al. (2013)	Luteolin	MCAO	SD rats	Luteolin(4 mg/kg) for 48 h	NA	Reduce neurological deficit (Longa) Reduce infarct size (TTC)	Caspase-3 path	Downregulated

**TABLE 3:** NHE therapeutic effects and mechanisms in *in vivo* ACI models.

Author(Year)	Extracts	Model	Cell types	Interventions	Positive control	Biological effects (experimental protocol)	Mechanism	Regulation
Liu et al. (2022)	NGR1	OGD	N2A	NGR1 (5/10/20/100/200 μM) for 24 h	Dl-3-n-Butylphthalide	Enhance cell viability (CCK-8) Improve mitochondrial dysfunction (mitochondrial membrane potential detection)	NA	
Liu et al. (2021a)	NGR1	OGD	bEnd.3	NGR1 (200 μM)	Dl-3-n-Butylphthalide	Protect the blood-brain barrier (Westernblot)	caveolin1/MMP2/9 path	Upregulated
Liu et al. (2021b)	SAA	OGD	HBMEC	SAA (10 μM)	aspirin	Enhance cell viability (CCK-8)	VEGFA/Src/VAV2/Rac1/PAK path	Downregulated
Zhang et al. (2023b)	SHPL-49	OGD	PC12	SHPL-49 (100/200 μM) for 24 h	NA	Inhibit calcium inflow (fluorescent probe) Enhance cell viability (CCK-8) Inhibit oxidative stress response (ROS assay) Inhibit apoptosis (Hoechst staining)	Bax/Bcl-2/Caspase-3 path	Downregulated
Li et al. (2015a)	T-VA	Model of cell injury induced by COCl_2_	PC12	T-VA (15/30/60 μM) for 36 h	NA	Enhance cell viability (MTT) Inhibit inflammatory response (immunohistochemical)	NF-κB/p65 COX-2	Downregulated
Jiang et al. (2018a)	celastro	OGD	Primary neurons、Primary microglia	Celastro (0.25/0.5/1/2 μM) for 3 h	NA	Enhance cell viability (CCK-8) Inhibit apoptosis (Flow Cytometry) Inhibit inflammatory response (Westernblot)	IL-33/ST2	Upregulated
Ferreira et al. (2023)	EDAC	OGD	Hippocampal slices	EDAC (1/10 μg/mL) for 1 h	NA	Alleviate glutamate hyperexcitation injury (Annexin V/PI assay) Protect astrocytes and oligodendrocytes (immunohistochemical)	Glutamate receptor	Downregulated
Sun et al. (2015)	Asiaticoside	OGD	Primary neurons	Asiaticoside (10/100 nM) for 24 h	NA	Enhance cell viability (MTT)	Bax/Bcl-2/Caspase-3 path	Downregulated
Zhang et al. (2013)	Luteolin	OGD	SH-SY5Y	Luteolin(10/25/50 ug/mL)	sulforaphane(10 μM)	Enhance cell viability (MTT)	Nrf2	Upregulated

### 3.2 CCI

We included 28 studies on CCI, including 28 *in vivo* and four *in vitro* studies. We summarized CCI-related research characteristics (Table 4). NHEs alleviated damage caused by CCI and improved cognitive dysfunction caused by ischemia (Table 5). Detailed information on four studies outlining *in vitro* CCI characteristics (extracts, interventions, biological effects, and mechanisms) is shown (Table 6).

**TABLE 4:** Summary of CCI study characteristics.

*In vivo*		Quantity	*In vitro*		Quantity
Model			Model		
	MCAO	2		OGD	3
	rUCCAO	1		Model of cell injury induced by H_2_O_2_	1
	2VO	14	Cell types		
	BCAS	3		PC12	1
	BCCAo	5		SH-SY5Y	1
	4VO	1		Primary neurons	1
	PBOCCA	2		HT-22	1
Species			Drug effect		
	SD rats	14		Enhance cell viability	1
	C57BL/6 mice	5		Inhibit apoptosis	1
	Wistar rats	9		Alleviate hypoxic damage	1
Drug effect				Ameliorate mitochondrial dysfunction	1
	Reduce neurological deficit	6			
	Induce proliferation of neural progenitor cells	1			
	Improve cognitive impairment	24			
	Inhibit activation and proliferation of astrocytes	2			
	Inhibit oxidative stress response	5			
	Inhibit apoptosis	1			
	Inhibit inflammatory response	3			
	Inhibit neuronal demyelination	3			
	Protect neuronal dendrites	1			
	Inhibit microglial activation	1			
	Improve regional cerebral blood flow disturbance	1			
	Reduce infarct size	1			

**TABLE 5:** NHE therapeutic effects and mechanisms in *in vivo* CCI models.

Author(Year)	Extracts	Model	Species	Interventions	Positive control	Biological effects (experimental protocol)	Mechanism	Regulation
Zhang et al. (2017b)	SA	MCAO	C57BL/6 mice	SA (15/30 mg/kg) for 14 d	NA	Reduce neurological deficit (mNSS) Induce proliferation of neural progenitor cells (Westernblot)	SHH/BDNF NGF	Upregulated
Wan et al. (2022)	Triptolide	rUCCAO	C57BL/6 mice	Triptolide (5/20 ug/kg) for 28 d	NA	Improve cognitive impairment (new object recognition test, Morris water maze)	Src/Akt/GSK3β path	Upregulated
Feng et al. (2012)	LIG	2VO	SD rats	LIG (80 mg/kg) for 7 d	NA	Improve cognitive impairment (Morris Water Maze) Inhibit activation and proliferation of astrocytes (immunohistochemical)	NA	
Peng et al. (2022a)	LIG	2VO	SD rats	LIG (20/40 mg/kg) for 28 d	NA	Improve cognitive impairment (Morris Water Maze) Inhibit oxidative stress response (MDA assay)	SIRT1/IRE1α/XBP1s/CHOP path	Upregulated
Yang et al. (2022a)	SAA	2VO	Wistar rats	SAA (5/10/20 mg/kg) for 56 d	nimodipine(10 mg/kg)	Improve cognitive impairment (Morris water maze, open field test) Inhibit apoptosis (TUNEL staining) Inhibit inflammatory response (immunofluorescence)	Drd2/Cryab/NF-κB path	Upregulated
Tan et al. (2022)	Que	BCAS	C57BL/6 mice	Que (60 mg/kg) for 14 d	NA	Improve cognitive impairment (Morris water maze, open field test, tail suspension test, forced swimming test, sucrose preference test)	NA	
Liu et al. (2019b)	CZ-7	2VO	Wistar rats	CZ-7 (10/20/40 mg/kg) for 25 d	nimodipine(20 mg/kg)	Improve cognitive impairment (Morris Water Maze) Inhibit oxidative stress response (MDA assay)	Nrf2	Upregulated
Zhang et al. (2023a)	Honokiol	BCAS	C57BL/6 mice	Honokiol (10 mg/kg) for 30 d	NA	Improve cognitive impairment (open field test, new object recognition test, fear conditioning, Y maze) Inhibit neuronal demyelination (immunohistochemical)	Akt/mTOR path	Upregulated
Chen et al. (2018a)	HAR	2VO	Wistar rats	HAR (15 mg/kg) for 60 d	NA	Improve cognitive impairment (Morris water maze, passive avoidance experiment)	PTEN/Akt/GSK3β	Downregulated
Lee et al. (2015)	Fructus extracts	BCCAo	Wistar rats	Fructus extracts (200 mg/kg) for 40 d	NA	Inhibit neuronal demyelination (immunohistochemical) Inhibit inflammatory response (Westernblot)	TLR4/MyD88 p38 MAPK	Downregulated
Li et al. (2012b)	Polydatin	4VO	SD rats	Polydatin (12.5/25/50 mg/kg) for 30 d	Ginkgo Tablets (25 mg/kg)	Improve cognitive impairment (Morris Water Maze) Inhibit oxidative stress response (MDA assay)	NA	
Shi et al. (2020)	Gas	2VO	SD rats	Gas (22.5/90 mg/kg) for 28 d	NA	Improve cognitive impairment (Morris water maze, attention diversion test)	NA	
Wu et al. (2023)	Gas	2VO	SD rats	Gas (25/50 mg/kg) for 28 d	NA	Improve cognitive impairment (Morris water maze, passive avoidance experiment) Reduce neuronal ischemic injury (immunohistochemical)	NA	
Yao et al. (2021)	EGB761	2VO	SD rats	EGB761(100 mg/kg)for 30 d	NA	Improve cognitive impairment (Morris water maze, new object recognition test) Inhibit neuronal demyelination (immunohistochemical)	mTOR	Upregulated
Kim et al. (2016)	GBE	BCCAo	Wistar rats	GBE (5/10/20/40 mg/kg) for 42 d	NA	Inhibit activation and proliferation of astrocytes (immunohistochemical) Inhibit inflammatory response (Westernblot)	NA	
Niu et al. (2020)	EF	2VO	SD rats	EF (50/100/200 mg/kg) for 84 d	nimodipine(10 mg/kg)	Improve cognitive impairment (new object recognition test, Y maze) Protect neuronal dendrites (immunohistochemical)	NRG1/ErbB4 BDNF/Fyn PI3K/Akt/CREB	Upregulated
Li et al. (2015b)	ICA	BCCAo	SD rats	ICA (10/40 mg/kg) for 23 d	NA	Improve cognitive impairment (Morris Water Maze)	BACE1 ADAM10 IDE	Downregulated(BACE1)、Upregulated(ADAM10、IDE)
Wan et al. (2017)	GSRd	BCAS	C57BL/6 mice	GSRd (10/30 mg/kg) for 21 d	NA	Improve cognitive impairment (Morris water maze, open field test) Reduce neuronal ischemic injury (HE staining)	BDNF	Upregulated
Zong et al. (2019)	CK	2VO	SD rats	CK (50/100/200 mg/kg) for 56 d	donepezil(2 mg/kg)	Improve cognitive impairment (Morris Water Maze) Reduce neuronal ischemic injury (HE staining)	GSK3β IDE	Upregulated(IDE) Downregulated(GSK3β)
Zhu et al. (2018)	GSRg1	2VO	Wistar rats	GSRg1 (50/100 mg/kg) for 56 d	nimodipine(20 mg/kg)	Improve cognitive impairment (Morris water maze, balance beam test) Reduce neuronal ischemic damage (Westernblot)	Bcl-2/Bax VEGF	Upregulated
Hwang et al. (2011)	SB extracts	BCCAo	Wistar rats	SB extracts (100/200 mg/kg) for 40 d	donepezil(10 mg/kg)	Improve cognitive impairment (Morris Water Maze)	MAPKs	Upregulated
Ahad et al. (2023)	CTRF	PBOCCA	SD rats	CTRF (10/20/40 mg/kg)	NA	Improve cognitive dysfunction (Morris water maze, passive avoidance test, open field test)	NA	
Damodaran et al. (2018)	CTRF	PBOCCA	SD rats	CTRF (100/200/300 mg/kg)	NA	Improve cognitive dysfunction (Morris water maze, passive avoidance test, open field test)	NA	
Tiang et al. (2020)	XEFGM α-MG	2VO	SD rats	XEFGM (25/50/100 mg/kg) for 40 d α-MG (25,50 mg/kg) for 40 d	NA	Improve cognitive dysfunction (Morris water maze, open field test)	NA	
Hosseinzadeh et al. (2012)	CSL extracts crocin	2VO	Wistar rats	CSL extracts (50/100/250 mg/kg) for 5 d Crocin (5/10/25 mg/kg) for 5 d	NA	Improve cognitive dysfunction (Morris water maze)	NA	
Kim et al. (2023)	AA	BCCAo	Wistar rats	AA (150/750 mg/kg) for 56 d	NA	Improve cognitive dysfunction (open field test, Y maze, eight-arm maze test) Inhibit microglial activation (immunohistochemical) Inhibit oxidative stress response (immunohistochemical)	Nrf2/Keap1/ARE path	Upregulated
Guang and Du. (2006)	pinocembrin	2VO	SD rats	Pinocembrin (0.5/5 mg/kg) for 14 d	NA	Improve regional cerebral blood flow disturbance (laser-Doppler) Improve cognitive impairment (Morris Water Maze) Inhibit oxidative stress response (Measurement of hydrogen peroxide production in mitochondria)	NA	
Zhou et al. (2021b)	PNS	MCAO	SD rats	PNS (72 mg/kg) for 14/28 d	nimodipine(14.4 mg/kg)	Reduce neurological deficit (Longa) Reduce infarct size (TTC)	ROCKII	Downregulated

**TABLE 6:** NHE therapeutic effects and mechanisms in *in vitro* CCI models.

Author(Year)	Extracts	Model	Cell types	Interventions	Positive control	Biological effects (experimental protocol)	Mechanism	Regulation
Peng et al. (2022a)	LIG	OGD	PC12	LIG (80 µM) for 2 h	NA	NA	SIRT1/IRE1α/XBP1s/CHOP path	Upregulated
Yang et al. (2022a)	SAA	OGD	SH-SY5Y	SAA (0.05/0.5/5/10/50 µM)	NA	Inhibit apoptosis (Flow Cytometry)	Drd2/Cryab/NF-κB path	Upregulated
Li et al. (2012b)	Polydatin	OGD	Primary neurons	Polydatin (12.5/5 μg/mL)	NA	Alleviate hypoxic damage (phase-contrast microscopy)	NA	
Wu et al. (2023)	Gas	Model of cell injury induced by H_2_O_2_	HT-22	Gas (100 µM)	NA	Enhance cell viability (MTT) Ameliorate mitochondrial dysfunction (oxygen consumption rate)	NA	

### 3.3 Cerebral IR

Of the 71 cerebral IR-related studies, 68 were *in vivo* and 31 were *in vitro* based. A summary of study characteristics is shown (Table 7), and then we describe the studies *in vivo* and *in vitro*, separately. Studies showed that NHEs had therapeutic roles in *in vivo* brain IR models (Supplementary Table S1). NHEs also reduced IR damage in *in vitro* models (Table 8).

**TABLE 7:** Summary of IR study characteristics.

*In vivo*		Quantity	*In vitro*		Quantity
Model			Model		
	MCAO/R	65		OGD/R	24
	4VO	1		Model of cell injury induced by H_2_O_2_	1
	2VO	2		Model of cell injury induced by glutamic acid	1
Species				EAN	1
	SD rats	41	Cell types		
	C57BL/6 mice	11		PC12	4
	Wistar rats	6		SH-SY5Y	5
	Swiss mice	1		Primary neurons	8
	ICR mice	7		BMEC	1
	Long-Evans rats	1		bEnd.3	2
	Trpm8^−/−^ mice	2		Primary microglia	7
	Mongolian gerbils	1		Primary cortical capillary endothelial cells	1
	Kunming mice	1		HUVEC	1
Drug effect				HBMEC	1
	Reduce neurological deficit	40		BV2	2
	Reduce cerebral edema	11		N2A	1
	Inhibit oxidative stress response	11		Olineu	1
	Reduce infarct size	45		C17.2 cell	1
	Inhibition inflammatory response	17	Drug effect		
	Facilitate the production of new neurons	3		Enhance cell viability	14
	Protect the blood-brain barrier	8		Inhibit apoptosis	5
	Inhibit neuronal autophagy	4		Inhibit granulocyte adhesion	1
	Improve regional cerebral blood flow disturbance	3		Promote endothelial cell proliferation, migration and invasion	1
	Increase angiogenesis	4		Inhibit oxidative stress response	5
	Promote astrocyte activation and proliferation	3		Protect the blood-brain barrier	1
	Inhibit degradation of tight junctions in ischemic areas	1		Inhibit degradation of tight junctions in ischemic areas	1
	Inhibit brain infiltration by NK cells	1		Inhibition inflammatory response	7
	Promote M2 microglia/macrophage polarization	1		Promote neuronal proliferation and differentiation	1
	Promote neurotrophic factor expression	1		Inhibit autophagy	1
	Improve cognitive impairment	3		Alleviate glutamate-induced neuronal damage	1
				Inhibit microglial activation	2
				Inhibit glutamate-induced calcium increase	1

**TABLE 8:** NHE therapeutic effects and mechanisms in *in vivo* IR models.

Author(Year)	Extracts	Model	Cell types	Interventions	Positive control	Biological effects (experimental protocol)	Mechanism	Regulation
Zhang et al. (2017a)	LHA	OGD/R	bEnd.3	LHA(15 uM)	NA	Inhibit degradation of tight junctions in ischemic areas (immunofluorescence)	HDAC4/NOX4/MMP-9 path	Upregulated
Zhang et al. (2018)	DGMI GA GB GC	OGD/R	PC12	DGMI(1/10/20 ug/mL) GA, GB or GC (10 μmol/L)	N-Acetyl-L-cysteine	Enhance cell viability (MTT) Inhibit oxidative stress response (Westernblot)	PI3K/Akt/Nrf2/HO-1 path PI3K/Akt/CREB/Bcl-2/Bax/Caspase-3 path	Upregulated
Yang et al. (2018)	EGB761 GB	EAN	Primary microglia Primary cortical capillary endothelial cells	EGB761 (0.1 mg/mL) GB (100 mmol/L)	NA	Enhance cell viability (MTT) Protect the blood-brain barrier (Na-F analysis) Inhibit apoptosis (Westernblot)	Bax/Bcl-2 path	Downregulated
Tu et al. (2018)	NGR1	OGD/R	Primary neurons	NGR1 (10 μM)	NA	Enhance cell viability (MTT)	ER/PI3K/Akt/mTOR JNK path	Upregulated
Ling et al. (2021)	SAA	OGD/R	Primary neurons Primary microglia	SAA (62.5/125/250 μg/mL) for 15 m	NA	Inhibit inflammatory response (Westernblot)	TLR2/4	Downregulated
Jiang et al. (2011)	SAA	OGD/R	BMEC	SAA (0.025/0.25/2.5/25 mg/L) for 20 h	NA	Inhibit granulocyte adhesion (cone-plate rheometer)	ICAM-1	Downregulated
Song et al. (2019)	SAA	OGD/R	SH-SY5Y	SAA (0.05/0.5/5 μM) for 24 h	NA	Enhance cell viability (MTT)	Akt/FOXO3a/BIM	Upregulated
Luan et al. (2020)	SA	OGD/R	PC12	SA (5 μM) for 24 h	Edaravone	Enhance cell viability (CCK-8) Inhibit oxidative stress response (MDA assay) Inhibit apoptosis (Hoechst staining)	Caspase-3 path	Downregulated
Zhang et al. (2019)	GSF1	OGD/R	HUVEC HBMEC	GSF1 (20/40 μM) for 4/8/12/24 h	VEGF(80 ng/mL)	Promote endothelial cell proliferation, migration and invasion (Transwell assays)	IGF-1/IGF1R path	Upregulated
Yuan et al. (2020)	PF11	OGD/R	Primary neurons	PF11 (30/100/200 μM) for 24 h	Dl-3-n-Butylphthalide	Promote neuronal proliferation and differentiation (BrdU administration)	BDNF/TrKB path	Upregulated
Zhang et al. (2020a)	GSRd	OGD/R	Primary neurons	GSRd (1/3/10/30/100 μM)	NA	NA	DAPK/NR2b/NMDAR	Downregulated
Zhao et al. (2021)	Betulinic Acid	OGD/R	PC12	Betulinic Acid	NA	Inhibit autophagy (Flow Cytometry)	SIRT1/FOXO1	Upregulated
Wang et al. (2007)	Emodin-8-O-beta-D-glucoside	Model of cell injury induced by glutamic acid	Primary neurons	Emodin-8-O-beta-D-glucoside (2.5/5/10 mg/kg) for 1 d	MK-801(10 uM)	Alleviate glutamate-induced neuronal damage	NA	
Leung et al. (2020)	emodin	OGD/R	PC12	Emodin (1/10 μM) for 4 h	NA	Inhibit oxidative stress response (ROS assay)	GLT-1 ERK-1/2/Bcl-2/Caspase-3	Upregulated
Yang et al. (2020)	Procyanidins	OGD/R	BV2	Procyanidins (10 μM)	NA	Inhibit inflammatory response (Westernblot)	TLR4/p38/NF-κB/NLRP3	Downregulated
Chen et al. (2020)	Glycyrrhizin	OGD/R	bEnd.3	Glycyrrhizin (10 μM)	rt-PA(20 ug/mL)	NA	ONOO-/HMGB1/TLR2/MMP9	Downregulated
Wang et al. (2019)	EK100	OGD/R	N2A	EK100 (20/40 μM)	NA	Inhibit apoptosis (Annexin V/PI assay)	p65 NF-κB Caspase-3	Downregulated
Li et al. (2021c)	ASIV	OGD/R	Primary microglia Primary neurons	ASIV (50 μM)	NA	Inhibit inflammatory response (Westernblot)	STAT3/CCL2	Downregulated
Mao et al. (2017)	Gas-d	Model of cell injury induced by H_2_O_2_	SH-SY5Y	Gas-d (10 uM) for 24 h	NA	Enhance cell viability (MTT) Inhibit oxidative stress response (ROS assay) Inhibit inflammatory response (ELISA)	NA	
Bai et al. (2024)	PQS	OGD/R	Primary microglia	PQS (25/100 ug/mL)	NA	Inhibit microglial activation (ELISA)	Nrf2/miR-103-3p/TANK	Upregulated
Zhang et al. (2024)	VOEX	OGD/R	Primary microglia	VOEX (6.25/12.5/25/50/100 μM) for 24 h	Dl-3-n-Butylphthalide(10uM)	Enhance cell viability (CCK-8)	IL17A	Downregulated
Zhou et al. (2021b)	PNS	OGD/R	SH-SY5Y	PNS (20/40/80/160/320/640 μg/mL)	NA	Enhance cell viability (CCK-8)	ROCKII	Downregulated
Qin et al. (2012)	PAL extracts	OGD/R	Primary neurons	PAL extracts (0.0156/0.0625/0.25 mg/mL)	NA	Enhance cell viability (MTT)	Caspase-9/3	Downregulated
Wan et al. (2022)	Triptolide	OGD/R	BV2 Olineu	Triptolide (0.001/0.01/0.1 nM) for 24 h	NA	Inhibit apoptosis (Hoechst staining) Inhibit inflammatory response (ELISA)	Src/Akt/GSK3β	Upregulated
Tan et al. (2022)	Que	OGD/R	Primary microglia	Que (30/60 µM) for 2 h	NA	Facilitate microglial phenotype switching (transmission electron microscopy) Inhibit inflammatory response (ELISA)	NA	
Wan et al. (2017)	GSRd	OGD/R	Primary neurons	GSRd (0.1/1/10 μM) for 2 h	NA	Enhance cell viability (MTT)	BDNF	Upregulated
Damodaran et al. (2018)	CTRF	OGD/R	Primary neurons	CTRF (2 μg/mL) for 24 h	NA	Inhibit glutamate-induced calcium increase (Fura-2 calcium imaging)	NA	
Wang et al. (2023b)	ASIV	OGD/R	SH-SY5Y	ASIV(10/20/40 μM) for 24 h	Ferrostatin-1(10 μM) for 24 h	Enhance cell viability (CCK-8) Inhibit peroxidation (ROS assay)	Nrf2	Upregulated
Li et al. (2024)	Sophoricoside	OGD/R	Primary neurons	Sophoricoside(25/50 μM)	NA	Enhance cell viability (CCK-8) Inhibit apoptosis (Westernblot, RT-qPCR) Inhibit inflammatory response (Westernblot, RT-qPCR)	Bax/Bcl-2 pAMPK	Upregulated(pAMPK) Downregulated(Bax/Bcl-2)
Park et al. (2009)	ESF	OGD/R	SH-SY5Y	ESF(0.4/2/10 ug/mL)	NA	Enhance cell viability (MTT)	Caspase-3	Downregulated
Chen et al. (2019)	ASIV	OGD/R	C17.2 cell	ASIV(100 μM)	Gefitinib(10 nM)	Enhance cell viability (MTT)	EGFR/MAPK	Upregulated

## 4 Quality evaluation of selected studies

**FIGURE 2:**
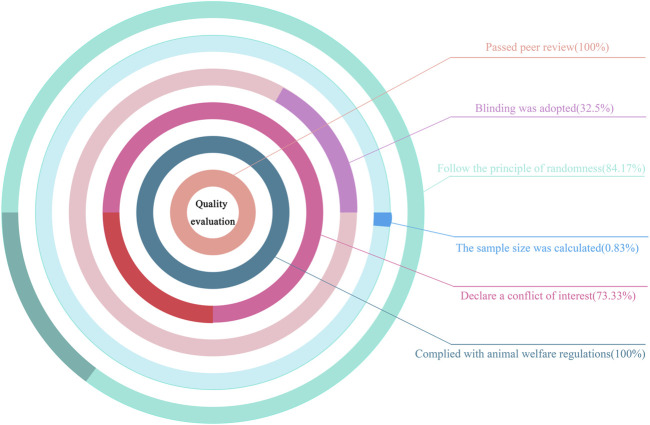
Quality evaluation chart.

Using our bias risk assessment scale, bias risk assessments were conducted on the 120 studies. All were peer-reviewed publications and they strictly complied with animal welfare regulations, which meant that all studies had at least two points (total score = 6 points). Additionally, 120 studies followed randomization principles (84.17%), 39 adopted blind methods (32.5%), 88 declared conflicts of interest among authors (73.33%), and only one study statistically calculated the sample size (0.83%) (Figure 2). Perhaps some researchers had calculated sample sizes before their studies, but this was not stated. After evaluations, bias risk scores for studies were in the 2–6 range: six studies scored 2 (5%), 29 scored 3 (24.17%), 56 scored 4 (46.67%), 28 scored 5 (23.33%), and one scored 6 (0.83%). Approximately half (46.67%) received four points, which proved that study quality was high. Bias risk evaluations for studies are shown (Table 7).

## 5 Toxicity

Unfortunately, many studies failed to provide NHE-related toxicity information. While drug toxicity studies are usually conducted at pre-experimental stages, researchers must articulate this. To complement the required NHE toxicity reports for this review, we performed additional NHE safety reviews by summarizing the toxicity reports in selected studies (Supplementary Table S2). However, not all NHEs have accompanying toxicity reports, which undoubtedly confirms a lack of safety studies in the natural herb research field. Reported that the LD50 (median lethal dose, the minimum amount of toxin required to kill half of an animal of a certain weight or age within a specified period of time) of SAA in mice (the dose required to kill half of cells/animal after a specific trial duration) was 1,161.2 mg/kg (Yang M.-Y. et al., 2022). *G. biloba* extracts may be carcinogenic and caution is recommended for their long-term use (Mei et al., 2017). ASIV is not toxic during maternal and embryonic development, but may inhibit fertility in female rats, suggesting its non-use in perinatal periods (Xuying et al., 2010). Luo et al. reported that emodin reduces and inhibits human sperm motility, suggesting some reproductive toxicity in these cells (Luo et al., 2015). LHA did not generate significant adverse reactions in toxicity tests in multiple experimental animals (Zhu et al., 2018). The main component of an ethanolic extract from *Erythrina velutina* Willd. (EEEV) is gallic acid, with *in vivo* studies showing that gallic acid at 210 mg/kg exerted no toxic effects in mice (Li et al., 2019). Ginkgolide A and ginkgolide B reduce mouse blastocyst viability and cause embryonic retardation in mice, leading to embryo death, suggesting caution when using these reagents during pregnancy (Mei et al., 2017). The main component of supercritical CO2 extracts from DanShen (SCED) is tanshinone IIA (TSA); it was found that at high TSA concentrations (25M), zebrafish embryo models exhibited severe growth inhibition, developmental malformations, and cardiotoxicity (Wang T. et al., 2017). Usually there are no obvious side effects when *Panax notoginseng* is supplemented to patients, but due to its estrogen effects, some patients have reported vaginal bleeding and distending breast pain. Those patients taking high *P*. *notoginseng* doses (>2.5 g/day) have central nervous system damaging effects such as insomnia, tachyarrhythmia, hypertension, and tension (Mancuso and Santangelo, 2017). *In vivo* betulinic acid studies showed no signs of systemic toxicity (Liu C. et al., 2019). Scutellarin has the lowest toxicity in rodents, and can even be said to be non-toxic. Quercetin (Que) toxicity is low; the organ weights and histopathology of rats treated with 400 mg/kg/d Que for 410 consecutive days showed no significant changes. Ginsenoside Rd, ginsenoside Rb1, and notoginsenoside promote cancer cell apoptosis and have significant effects in cancer treatment. Notoginsenoside R1 also inhibits cell proliferation, migration, invasion and angiogenesis, and promotes cell apoptosis at 150 µM. Shikonin is considered safe, but may cause skin allergies at very low doses. Luteolin exerts cytotoxicity at 5 μM and 10 µM doses, and its safety must be further evaluated in animal models and clinical trials. Hydroxysafflor yellow A is sensitive to interactions between herbs and drugs, resulting in no therapeutic effects at certain doses (Guo et al., 2023). Galangin has an IC50 (half-inhibitory concentration, the concentration at which a biological process or activity is inhibited by 50%) value of 275.48 μM in V79 cells, and does not produce genotoxic effects at all concentrations (Bacanlı et al., 2017). Similarly, echinocystic acid (EA) exerts no cytotoxic effects under any conditions in cell viability assays (Joh et al., 2012). Glycyrrhizin is moderately toxic and should be used with caution during pregnancy. It also has selective cytotoxic effects toward cancer cells, and its most important side effects are secondary diseases induced by hypertension and hypokalemia (Nazari et al., 2017). After the maternal application of 1.0 mg/kg ASIV for 28 consecutive days, ASIV delays development in young rats and should be used with caution in perinatal women (Zhang J. et al., 2020). Studies report serious adverse reactions to matrine, the most serious being hepatotoxicity, neurotoxicity, and reproductive toxicity (Wang X. et al., 2023). Acute and subacute toxicity studies report that breviscapine is a safe drug with a potential for widespread use in clinical settings (Wu et al., 2021). Icariin has an IC50 of 20 μM in HeLa cells, and its toxic effects in normal cells are relatively negligible (Huang et al., 2019).

## 6 Discussion

**FIGURE 3:**
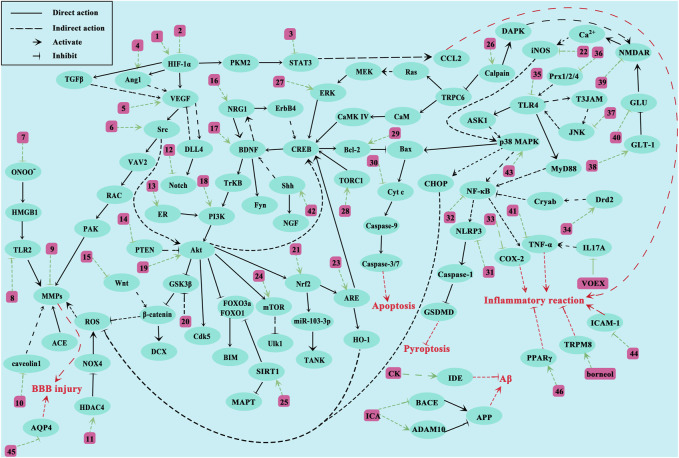
Proposed NHE molecular mechanisms for treating CI. The green line represents the interaction between NHEs and molecules. The red line represents the pathological effects caused by molecules. Numbers represent NHEs (1, DL-NBP; 2, PNS, SAA and SA; 3, ASIV and Formononetin; 4, L-borneol; 5, T-VA and GSRb1; 6, Triptolide; 7, Glycyrrhizin and SAA; 8, SAA; 9, ASIV and Methylophiopogonanone A; 10, NGR1; 11, LHA; 12, L-borneol; 13, NGR1; 14, HAR; 15, L-borneol; 16, EF; 17, GSRd, LHA, PF11, and SAA; 18, EK100, TFCJ, DGMI, and Formononetin; 19, Honokiol, L-NBP, HSYA, and GSF1; 20, CK; 21, Catalpol, KRGP, CZ-7, AA, Rus, PQS, and ASIV; 22, Honokiol; 23, EGB761; 24, EGB761 and Vitexin; 25, LIG, BA and ASIV; 26, Hyperforin and GSRd; 27, emodin; 28, TSA; 29, Catalpol, MO, SHPL-49, Galangin, KuA, Asiaticoside, GSRb1, OLE, EGB761, SA, GB, Sophoricoside and Matrine; 30, PAL extracts and GSRb1; 31, SB extracts; 32, T-VA, Silymarin, Storax, EK100 and Rus; 33, ES; 34, SAA; 35, Fructus extracts, Procyanidins and SAA; 36, Gas-d; 37, YZR extracts and EA; 38, GSRb1 and emodin; 39, GSRb1 and GSRd; 40, EEEV, scutellarin, EDAC and CTRF; 41, MO; 42, SA and PNS; 43, L-borneol, SB extracts and ASD extract; 44, ASIV and Vitexin; 45, GSRg1, ASIV and DSE; 46, Silymarin, SAA and ICA).

We comprehensively summarized the molecular mechanisms underlying CI treatment by NHEs (Figure 3). As mentioned, NHE therapeutic effects toward CI are roughly divided in two ways: one reduces damage, mainly by improving local blood flow disturbance, inhibiting oxidative stress, inflammatory responses and apoptosis, relieving cerebral edema, protecting the BBB, and inhibiting excitatory amino acid overexpression. The other way promotes injury recovery, mainly by promoting endothelial cell proliferation and migration, promoting neuron proliferation and differentiation, and promoting neurotrophic factor expression. NHEs may also act on molecules, such as SAA, *via* several pathways. SAA is a bioactive compound extracted from *Salvia miltiorrhiza Bunge*. Studies report that SAA has direct or indirect effects on toll-like receptor 2/4 (TLR2/4), phosphoinositide 3-kinase (PI3K), glycogen synthase kinase 3β (GSK3β), vascular endothelial growth factor A (VEGFA), and intercellular adhesion molecule 1 (ICAM1), which means that SAA not only inhibits apoptosis, inflammation, and oxidative stress, and protects the BBB, but also promotes vascular proliferation and recovery (Jiang et al., 2011; Chien et al., 2016; Song et al., 2019; Liu C. et al., 2021; Ling et al., 2021). Additionally, some key factors involved in multiple pathways, such as VEGF, are activated by multiple NHEs. The discovery of VEGF has completely changed our understanding of blood vessel production during development and physiological homeostasis. The biological effects mediated by VEGF are mainly due to its impact on vascular permeability and new blood vessel generation. VEGF has important relationships with tumor growth and metastasis, hypertensive retinopathy, and other pathological conditions (Apte et al., 2019). T-VA is extracted from *Ligusticum sinense* (Li G. et al., 2015). Several studies report that T-VA, ginsenoside Rb1 (GSRb1), L-borneol, and DL-n-butylphthalide (DL-NBP) can also play a role through VEGF (Li G. et al., 2015; Zhu et al., 2018; Ma et al., 2021; Wang et al., 2020).

CI is a severe nerve injury caused by interrupted cerebral blood flow. The molecular mechanisms underpinning its pathological processes are extremely complex and cannot be fully explained at present. CI involves amino acid excitation, injury oxidative stress, inflammatory responses, BBB injury, mitochondrial dysfunction, cell necrosis, and apoptosis. Many studies report that key molecules are involved in these CI-mediated processes, such as hypoxia-inducible factor alpha (HIF-1α), VEGF, brain-derived neurotrophic factor (BDNF), protein kinase B (Akt), matrix metalloproteinases (MMPs), c-Jun N-terminal kinase (JNK), B-cell lymphoma-2-associated X (Bax), Caspase-3/9, mitogen-activated protein kinases (MAPKs) and nuclear factor kappa-B (NF-κB) (Wang et al., 2020; Li et al., 2022; Liu et al., 2023; Li et al., 2017; Chen H.-S. et al., 2018; Hu et al., 2020; Peng T. et al., 2022; Xu et al., 2021).

Blood flow disturbance is the most fundamental issue in CI, and appears to initiate several pathological conditions, such as BBB damage, mitochondrial dysfunction, cell necrosis, and apoptosis. To reduce the severity and prognosis of stroke onset, doctors must rapidly conduct clinical interventions such as intravenous thrombolysis and surgical thrombectomy to unblock cerebral blood vessels. Anticoagulant and antiplatelet therapies are recommended for patients without contraindications, but their harsh conditions of use and severe side effects have prompted scientists to explore better treatments. Thromboxane A2 (TXA2) is a potent vasoconstrictor and the main cyclooxygenase (COX) product of arachidonic acid (AA). The functional importance of this eicosanoid in acute coronary ischemic syndrome has been demonstrated as it activates platelets (Reilly and Fitzgerald, 1993). Fei et al. extracted natural compounds with TSA as the main component from *S*. *miltiorrhiza Bunge* (DanShen); this SCED inhibits platelet aggregation and improves regional blood flow disorders by inhibiting TXA2 activation (Fei et al., 2017). Although the specific molecular mechanisms have not been clarified, and to determine NHE biological effects on blood flow disorders, several studies have used laser speckle imaging to show that *P*. *notoginseng* saponins (PNS), GSRb1, L-borneol, and galangin can improve regional blood flow disorders after stroke (Li S. et al., 2012; Wang S. et al., 2017; Gao et al., 2022; Xie et al., 2023).

Amino acid excitotoxicity is due to the abnormal accumulation of some excitatory amino acids (such as glutamate) outside neurons after ischemia. Glutamate accumulation leads to sustained Ca^2+^ channel and N-methyl-D-aspartic acid receptor (NMDAR) activation on neuronal synaptic membranes. NMDAR is an ion channel regulated by glutamate on cell membranes. After glutamate activation, high Ca^2+^ levels are transported into membranes. Ca^2+^ accumulates in the cytoplasm and mitochondria, resulting in Ca^2+^ overload. This alteration affects many biological processes, such as calpain activation, oxidative stress responses, and mitochondrial damage, and also protease, kinase, phosphatase, and other enzyme activities, leading to cell death. As a major transporter of excitatory amino acids, glutamate transporter 1 (GLT-1) is mainly distributed in astrocytes. Usually, GLT-1 mediates glutamate uptake by glial cells to maintain extracellular glutamate concentrations. GLT-1 function is impaired during CI, resulting in high intersynaptic glutamate accumulation (Rao et al., 2001). GSRb1 and emodin reportedly activate GLT-1 receptors on astrocyte membranes, transferring glutamate into astrocytes to reduce its abnormal accumulation outside neurons (Wang S. et al., 2017; Leung et al., 2020). GSRb1 also has the same effects as ginsenoside Rd (GSRd) in inhibiting NMDAR expression (Zhang C. et al., 2020). Glutamine synthetase (GS) catalyzes glutamate conversion to glutamine *in vivo* and has important roles regulating glutamate levels. KRGP is an active substance extracted from Korean red ginseng(Liu L. et al., 2019). Liu et al. found that GS expression levels are significantly elevated after CI in KRGP-pretreated mice, while GS expression levels are not changed much in nuclear factor erythroid 2-related factor 2 (Nrf2) gene deletion mice, suggesting that the Nrf2 pathway has important roles in glutamate homeostasis after CI, and that KRGP may reduce amino acid excitation damage caused by CI *via* Nrf2 signaling (Liu L. et al., 2019).

Oxidative stress is a common phenomenon in hypoxic cells. Mitochondria are essential organelles which maintain energy homeostasis in cells. The state and function of mitochondria undergo significant changes during hypoxia, leading to increased intracellular reactive oxygen species (ROS) levels, which severely damage cells and brain tissue. Oxidative stress products directly attack biomacromolecules (amino acids and nucleic acids) to induce apoptosis and increase BBB permeability. Nrf2 has antioxidant and anti-inflammatory effects that activate heme oxygenase-1 (HO-1) after oxidative stress-inducer (e.g., inflammatory chemokines/cytokines) activation or tissue damage (Hassanein et al., 2023). HO-1 is an inducible homolog with antioxidant properties and has important roles regulating oxidative stress, with elevated HO-1 levels detected in almost all oxidatively stressed cells. catalpol, KRGP, CZ-7, AA, and ruscogenin (Rus) increase HO-1 expression by stimulating Nrf2 (Liu L. et al., 2019; Liu D.-D. et al., 2019; Wang et al., 2022; Zhang S. et al., 2023; Kim et al., 2023). Additionally, by detecting mitochondrial energy metabolism-related genes (*Atp12a* and *Atp6v1g3*), Liu et al. found that notoginsenoside R1 (NGR1) mitigates mitochondrial energy metabolism abnormalities (Liu et al., 2022). Three Nei-like DNA glycosylases exist in mammalian cells, which protect DNA by removing oxidative bases. GSRd protects neurons by activating Nei-like DNA glycosylase 1/3 (NEIL1/3) to promote DNA hydrolysis of oxidative stress-induced product damage (Yang et al., 2016).

Inflammatory responses are self-defense mechanisms; they are stimulated by endogenous and exogenous inflammatory factors and are closely related to different diseases. Neuroinflammation occurs at almost all stages of ischemic stroke and is caused by damage-associated molecular pattern release by damaged/dead cells. These patterns, including adenosine, heat shock proteins, and interleukin 33 (IL-33) are recognized by corresponding immune cells which trigger multiple downstream signaling pathways (Qin et al., 2022). Additionally, these patterns stimulate inflammation-related cytokine, interferon or chemokine production, leading to increased adhesion molecule expression, helping white blood cells adhere to blood vessel surfaces, and promoting immune cell infiltration. Therefore, for patients with CI, early anti-inflammatory treatment is an important method to reduce ischemic injury and improve prognosis. Pro-inflammatory cytokines induce chemokine secretion immediately after CI. Chemokine-chemokineligand2 (CCL2) and its receptors are involved in regulating inflammation in the ischemic state, and may be recruited to and adhere to cerebral vascular endothelial cells by immune cells. Signal transducer and activator of transcription 3 (STAT3) has positive regulatory effects on chemokines (such as CCL2) and acts as a key transcription factor during inflammation and immunity. Li et al. reports that ASIV inhibits CCL2 functions by inhibiting STAT3 expression and inhibiting NK cell infiltration (Li S. et al., 2021). JNK, TLR4, NF-κB, and MAPKs also have key roles in inflammatory signaling pathways *via* a vicious cycle between JNK and TLR4 (Cheng et al., 2021a). TLRs are expressed on cell surfaces and in intracellular spaces, and regulate the state and function of many immune cells. Fructus extracts, procyanidins, and SAA inhibit TLR4 expression (Lee et al., 2015; Yang et al., 2020; Ling et al., 2021), while *Alpinia oxyphylla* Miq. (YZR) extract and EA inhibit JNK activation (Yu et al., 2019; Cheng et al., 2021a). T-VA, silymarin, storax, and EK100 also suppress inflammatory responses by inhibiting NF-κB (Hou et al., 2010; Li G. et al., 2015; Wang et al., 2019; Zhou M. et al., 2021). The MAPK signaling pathway is activated shortly after ischemic injury onset. MAPK is composed of three major effectors, extracellular signal-related kinases (ERK1/2), JNK, and p38 MAPK. Among these, p38 MAPK regulates pro-inflammatory cytokine expression. The activation of MAPK/ERK signaling and the stimulating effects of MMP expression can aggravate BBB injury in ischemic stroke and further enhance pro-inflammatory factor expression. Interestingly, we found that different NHEs have opposite effects on p38 MAPK, but all were protective against CI injury, which we speculate might be due to the activation of different factors downstream of p38 MAPK. L-borneol and *Angelica sinensis* (Oliv.) Diels (ASD) extracts activate p38 MAPK (Cheng et al., 2021b; Xie et al., 2023), while Honokiol and *Scutellaria baicalensis* Georgi (SB) extracts inhibit its function (Hwang et al., 2011; Chen et al., 2014).

MMPs are essential for BBB function and structure, and mainly act on the tight junction component, ZO-1, between adjacent cells. Endothelial cells and their tight junction components are key factors maintaining BBB stability. MMPs disrupt the BBB *via* enzymatic ZO-1 hydrolysis, so they are potential therapeutic targets for CI (Batra et al., 2010). Glycyrrhizin indirectly inhibits MMPs by reducing peroxynitrite (ONOO^−^) production (Chen et al., 2020). NGR1 mitigates BBB disruption by MMPs by inhibiting caveolin 1 (Liu B. et al., 2021). LHA promotes histone deacetylase 4 (HDAC4) expression, leading to decreased NADPH oxidase 4 (NOX4) expression, which in turn inhibits MMP expression (Zhang Q.-Y. et al., 2017). Additionally, ginsenoside Rg1 (GSRg1), ASIV, and DSE prevent ischemic cerebral edema and BBB damage by inhibiting AQP4 (Lee K. et al., 2012; Li et al., 2013; Zhou et al., 2014).

Apoptosis is a normal physiological activity, but after CI, the process becomes overactivated and causes neuronal death, which leads to neurological deficits in patients with CI, and seriously affects neurological function recovery in later stages. Bax is a classical apoptosis-promoting gene that promotes cytochrome C (Cyt-C) transfer from the mitochondria to cells, and then activates the caspase cascade to eventually lead to apoptosis. B-cell lymphoma-2 (Bcl-2) is an apoptosis inhibitor protein, which binds to Bax and forms dimers to inhibit apoptosis. Therefore, the balance between Bcl-2 and Bax is key to neuronal survival. Several NHEs protect neurons from apoptosis, such as catalpol, MO, SHPL-49, galangin, KuA, asiaticoside, GSRb1, oleuropein (OLE), EGB761, salvianolic acid (SA), and GB, by increasing Bcl-2 levels (Li S. et al., 2012; Sun et al., 2015; Yu et al., 2016; Liu et al., 2017; Zhao et al., 2017; Yang et al., 2018; Zhang et al., 2018; Zhu et al., 2018; Luan et al., 2020; Wang et al., 2022; Zhang P. et al., 2023). Additionally, SAA also inhibits neuronal apoptosis *via* the Akt/FOXO3a/BIM pathway (Song et al., 2019).

## 7 Conclusion and prospects

With deepening research on NHEs, their mechanisms are becoming more complex and multifaceted. Complexity means that a single NHE, such as SAA, can simultaneously act on multiple molecular pathways. Multifaceted means that a NHE acting on the same key factor on a certain pathway may have different regulatory outcomes, such as L-borneol and Honokiol. In such cases, a deeper understanding of study conditions and results is required. Although too many pathways and factors are involved in these studies, key factors such as VEGF, BDNF, Akt, MMPs, JNK, Bax, Caspase-3/9, MAPKs, and NF-κB, may provide reference points for further research. Additionally, although CI pathogenesis is highly complex, the first and most important pathology is disturbed blood flow, which is why early CI treatments should rapidly restore blood supply. Therefore, more attention should be paid to NHEs (TSA or GSRb1) with antiplatelet or antithrombotic effects. For advanced CI treatments, selected studies should mainly focus on inhibiting neuroinflammation, inhibiting neuronal apoptosis, and protecting the BBB. This means that NHE treatment effects for CI are significant, and show how important neuroinflammation and neuronal apoptosis processes are in CI. In addition, the treatment of transient cerebral ischemia and permanent cerebral ischemia is also different. Transient cerebral ischemia is a sudden, transient cerebral vascular insufficiency, usually without brain tissue necrosis. The treatment of transient cerebral ischemia is mainly prevention, such as antiplatelet therapy. Permanent cerebral ischemia often has abnormal pathology such as thrombus, which leads to blood flow interruption and eventually brain cell death. If the vascular recanalization treatment cannot be carried out in time, serious sequelae will often be left.

Although strict review conditions were set, some flaws were identified in selected studies: 1) The elaboration of extraction protocols or NHE sources was not adequately detailed. Due to different NHE extraction methods, resultant NHEs may have different biological activities and effects, which makes the research data unreliable; 2) NHE toxicity was not adequately explored in selected studies. Even if some studies performed toxicity tests, they were *in vitro* and not *in vivo*; 3) Although considerable animal data were observed, it is uncertain if these NHEs can be eventually used in clinical practice; therefore, more pharmacological and pharmacological studies are required; and 4) NHE mechanisms were not fully explored. Most studies only explored one or several related factors, but did not examine complete NHE mechanisms.

Clinically observed NHE effects are the result of combined drug actions across a multitude of signaling pathways. Researchers are constantly exploring new treatment options to maximize treatment benefits while minimizing side effects. Traditional Chinese medicine efforts in this area are worthy of recognition, and the synergistic actions of multiple NHEs should be considered in future research. In addition, the reproducibility of drug efficacy is very critical, and it is an important factor affecting whether a drug can be transformed into clinical practice. In order to do this, we need to maintain a rigorous working attitude and record the experimental process in detail. We should strengthen our understanding of the various parts of the experiment and conduct sufficient pre-experiments. The factors affecting the transformation of medical achievements also include the lack of advanced medical equipment, insufficient attention to medical transformation, and lack of communication between the supply and demand sides.

In this review, we retrieved and screened high-quality NHE studies related to CI. We briefly summarized the potential therapeutic effects and mechanisms underpinning NHEs toward CI, which may promote NHE development and their applications in clinical settings. Selecting a clinical medication is a long and complicated process, and any possibilities, to combat CI, must be carefully and comprehensively considered.

## Figures and Tables

**Table:** 

**CI**	cerebral ischemia
**ACI**	acute cerebral ischemia
**CCI**	chronic cerebral ischemia
**IR**	ischemia-reperfusion
**BBB**	blood-brain barrier
**NHE**	natural herbal extract
**rt-PA**	recombinant tissue plasminogen activator
**LHA**	*Leonurus japonicus* Houtt. total alkaloids
**KuA**	kukoamine A
**ICA**	icariin
**ASIV**	astragaloside IV
**SAA**	salvianolic acid A
**BA**	betulinic acid
**LIG**	ligustilide
**GB**	ginkgolide B
**TSA**	tanshinone IIA
**SCED**	supercritical CO_2_ extracts from *DanShen*
**NGR1**	notoginsenoside R1
**GSRb1**	ginsenoside Rb1
**GSRg1**	ginsenoside Rg1
**GSF1**	ginsenoside F1
**GSRd**	ginsenoside Rd
**PF11**	pseudoginsenoside-F11
**SA**	salvianolic acid
**Que**	quercetin
**HAR**	harpagoside
**GBE**	*Ginkgo biloba* L. extract
**EF**	epimedium flavonoids
**SB**	*Scutellaria baicalensis* Georgi
**Gas**	gastrodin
**CTRF**	C*litoria ternatea* L. root extract
**XEFGM**	xanthone-enriched fraction of *Garcinia mangostana* L.
**CSL**	*Crocus sativus* L.
**EEEV**	ethanolic extract from *Erythrina velutina* Willd.
**YZR**	*Alpinia oxyphylla* Miq.
**GA**	ginkgolide A
**GC**	ginkgolide C
**OLE**	oleuropein
**PQS**	*Panax quinquefolius* L. saponins
**VOEX**	*Verbena officinalis* L. ethanol extracts
**Rus**	ruscogenin
**TFCJ**	total flavonoids of *Chuju*
**NBP**	n-butylphthalide
**L-NBP**	L-n-butylphthalide
**DL-NBP**	DL-n-butylphthalide
**GL**	*Ganoderma lucidum*
**HSYA**	Hydroxysafflor Yellow A
**PNS**	*Panax notoginseng* saponins
**ES**	*Eleutherococcus senticosus*
**GRex**	*Glycyrrhiza uralensis* Fisch. methanolic extracts
**EO**	*Euterpe oleracea* Mart.
**ASD**	*Angelica sinensis* (Oliv.) Diels
**ESF**	EtOAc extract of *Sophora flavescens* Aiton
**MCAO**	middle cerebral artery occlusion
**PT**	photothrombosis
**rUCCAO**	right unilateral common carotid artery occlusion
**2VO**	bilateral carotid artery ligation
**BCAS**	bilateral common carotid artery stenosis
**BCCAo**	bilateral common carotid artery occlusion
**4VO**	4-vessel occlusion
**PBOCCA**	permanent bilateral occlusion of common carotid arteries
**MCAO/R**	middle cerebral artery occlusion and reperfusion
**mNSS**	modified Neurological Severity Score
**EB**	evans blue
**OGD**	oxygen-glucose deprivation
**OGD/R**	oxygen-glucose deprivation/re-oxygenation
**Nrf2**	nuclear factor erythroid 2-related factor 2
**HO-1**	heme oxygenase-1
**Bax**	B-cell lymphoma-2-Associated X
**Bcl-2**	B-cell lymphoma-2
**6-keto-PGF1α**	6-keto Prostaglandin F1α
**TXB2**	thromboxane B2
**TXA2**	thromboxane A2
**PAFR**	platelet-activating factor receptor
**PLC**	phospholipase C
**PKC**	protein kinase C
**TORC1**	target of Rapamycin Complex 1
**CREB**	cAMP-response element binding protein
**BDNF**	brain-derived neurotrophic factor
**VEGF**	vascular endothelial growth factor
**VEGFA**	vascular endothelial growth factor A
**Src**	tyrosine kinase Src
**VAV2**	vav guanine nucleotide exchange factor 2
**Rac**	Ras-related C3 botulinum toxin substrate
**PAK**	p21 activated kinase
**MMPs**	matrix metalloproteinases
**MMP2/9**	matrix metalloproteinase 2/9
**HIF-1α**	hypoxia-inducible factor alpha
**PKM2**	pyruvate kinase isozyme type M2
**STAT3**	signal transducer and activator of transcription 3
**GLT-1**	glutamate transporter 1
**NMDAR**	N-methyl-D-aspartic acid receptor
**NR2b**	N-methyl-D-aspartic acid receptor 2b
**Cyt-C**	cytochrome C
**NEIL1/3**	Nei Like DNA Glycosylase 1/3
**Ang1**	Angiopoietin-1
**NF-κB**	nuclear factor kappa-B
**COX-2**	cyclooxygenase-2
**IL-33**	interleukin 33
**IL-17A**	interleukin 17A
**ICAM1**	intercellular adhesion molecule 1
**ST2**	growth stimulation expressed gene 2
**SHH**	Sonic hedgehog
**NGF**	nerve growth factor
**Drd2**	dopamine D2 receptor
**Cryab**	αB-crystallin
**Akt**	protein kinase B
**GSK3β**	glycogen synthase kinase 3β
**SIRT1**	sirtuin 1
**IRE1α**	inositol-requiring enzyme-1α
**XBP1s**	X-box binding protein 1
**CHOP**	C/EBP-homologous protein
**mTOR**	mammalian target of rapamycin
**PTEN**	phosphatase and tensin homolog
**TLR4**	toll-like receptor 4
**TLR2**	toll-like receptor 2
**MyD88**	myeloid differentiation factor 88
**MAPK**	mitogen-activated protein kinase
**p38 MAPK**	p38 mitogen-activated protein kinase
**NRG1**	neuregulin1
**ErbB4**	epidermal growth factor receptor
**PI3K**	phosphoinositide 3-kinase
**BACE1**	beta-secretase 1
**ADAM10**	a disintegrin and metalloproteinase domain 10
**IDE**	insulin-degrading enzyme
**Keap1**	kelch-like ECH-associated protein 1
**ARE**	antioxidant response element
**ROCKII**	Rho-associated protein kinase II
**ONOO^-^**	peroxynitrite
**TrKB**	tyrosine kinase receptor B
**HDAC4**	histone deacetylase 4
**NOX4**	NADPH oxidase 4
**HPLC**	high performance liquid chromatography
**JNK**	c-Jun N-terminal kinase
**T3JAM**	TRAF3-interacting JNK-activating modulator
**ER**	estrogen receptor
**RIPK1/3**	receptor-interacting protein kinase 1/3
**NLRP3**	NOD-like receptor family pyrin domain-containing 3
**HMGB1**	high mobility group box 1
**Dll4**	delta-like ligand 4
**TRPC6**	transient receptor potential channel 6
**IGF-1**	insulin-like growth factor 1
**IGF1R**	insulin-like growth factor 1 receptor
**DAPK**	death-associated protein kinase
**ERK1/2**	extracellular signal-related kinases 1/2.

## Data Availability

The original contributions presented in the study are included in the article/Supplementary Material, further inquiries can be directed to the corresponding author.
