# Supplementary material for: Natural herbal extract roles and mechanisms in treating cerebral ischemia: A systematic review
Source: Front Pharmacol. 2024 Aug 2;15:1424146. doi: 10.3389/fphar.2024.1424146 (PMC11327066; doi:10.3389/fphar.2024.1424146)
Supplement: Supplementary file 1 [file Table1.docx]

**Supplementary Table 1. NHE therapeutic effects and mechanisms in *in vivo* cerebral IR models**

| Author(Year) | Extracts | Model | Species | Interventions | Positive control | Biological effects (experimental protocol) | Mechanism | Regulation |
| --- | --- | --- | --- | --- | --- | --- | --- | --- |
| Li et al.  (2021a) | LHA | MCAO/R | SD rats | LHA(25/50/100mg/kg) for 14d | NA | Inhibit inflammatory response (RT-qPCR)  Reduce infarct size (TTC)  Protect the blood-brain barrier (EB test) | BDNF/TrKB/PI3K/Akt path | Upregulated |
| Zhang et al.  (2017a) | LHA | MCAO/R | SD rats | LHA(15mg/kg) | NA | Reduce neurological deficit (neurological deficit scores)  Reduce infarct size (TTC)  Protect the blood-brain barrier (EB test)  Inhibit degradation of tight junctions in ischemic areas (immunofluorescence) | HDAC4/NOX4/MMP-9 path | Upregulated |
| Rodrigues et al.  (2017) | EEEV | 2VO | Swiss mice | EEEV(200/400mg/kg)for 5d | Memantine(10mg/kg)for 5d | Improve cognitive dysfunction (open field test, rotary rod test, Y maze test, object recognition test) | Amino acid | Downregulated(Excitatory amino acid)  Upregulated(Inhibitory amino acid) |
| Cheng et al.  (2021a) | YZR extracts | MCAO/R | SD rats | YZR extracts(200/400/800mg/kg) | NA | Reduce neurological deficit (mNSS)  Reduce infarct size (TTC)  Inhibit inflammatory response (immunohistochemical) | JNK-mediated TLR4/T3JAM path and ASK1 path | Downregulated |
| Zhang et al.  (2018) | DGMI | MCAO/R | SD rats | DGMI(1/3/10mg/kg) | Edaravone(10mg/kg) | Reduce neurological deficit (neurological deficit scores)  Alleviate ischemic brain edema (water content calculation)  Inhibit oxidative stress response (immunofluorescence)  Reduce infarct size (TTC) | Akt/Nrf2/HO-1 path  Akt/CREB path | Upregulated |
| Saleem et al.  (2008) | EGB761 | MCAO/R | C57BL/6 mice | EGB761(100mg/kg)for 7d | NA | Reduce neurological deficit (neurological deficit scores)  Reduce infarct size (TTC); | HO-1 | Upregulated |
| Yu et al.  (2016) | OLE | MCAO/R | ICR mice | OLE(100 mg/kg) | NA | Reduce neurological deficit (neurological deficit scores)  Reduce infarct size (TTC) | Bax/Bcl-2/Caspase-3 path | Downregulated |
| Tu et al.  (2018) | NGR1 | MCAO/R | SD rats | NGR1(15mg/kg) for 2d | NA | Reduce cerebral edema (water content calculation)  Reduce infarct size (TTC) | ER/PI3K/Akt/mTOR  JNK path | Upregulated |
| Ling et al.  (2021) | SAA | MCAO/R | SD rats | SAA(5/10mg/kg) for 7d | Edaravone(3mg/kg) | Alleviate ischemic brain edema (water content calculation)  Inhibit inflammatory response (immunofluorescence) | TLR2/4 | Downregulated |
| Chien et al.  (2016) | SAA | MCAO/R | ICR mice | SAA(0.05/0.1mg/kg) for 7d | rt-PA(10mg/kg) | Protect the blood-brain barrier (EB test)  Inhibit oxidative stress response (Westernblot)  Reduce infarct size (TTC) | PI3K/Akt  p25/Cdk5  GSK3β/Bcl-2  GSK3β/DCX | Upregulated(PI3K/Akt，p25/Cdk5)  Downregulated(GSK3β/Bcl-2,GSK3β/DCX) |
| Song et al.  (2019) | SAA | MCAO/R | SD rats | SAA(5/10/20mg/kg) for 3d | NA | Reduce infarct size (TTC) | Akt/FOXO3a/BIM | Upregulated |
| Luan et al.  (2020) | SA | MCAO/R | SD rats | SA(10/20/30mg/kg) for 3/14d | Edaravone(30mg/kg) | Reduce neurological deficit (Longa)  Protect the blood-brain barrier (EB test)  Inhibit apoptosis (TUNEL staining)  Reduce infarct size (TTC)  Improve long-term learning and memory defects (Morris water maze experiment) | Caspase-3 path | Downregulated |
| Zhang et al.  (2019) | GSF1 | MCAO/R | SD rats、Wistar rats | GSF1(50mg/kg) for 14d | NA | Increase angiogenesis (tube formation assay, CAM assay) | NA |  |
| Zhou et al.  (2014) | GSRg1 | MCAO/R | SD rats | GSRg1(20mg/kg) for 6h/1/3/7/14d | acetazolamide(100mg/kg) | Protect the blood-brain barrier (EB test) | AQP4 | Downregulated |
| Yuan et al.  (2020) | PF11 | MCAO/R | C57BL/6 mice | PF11(8/16/32mg/kg) for 7d | Dl-3-n-Butylphthalide(40mg/kg) | Reduce cerebral edema (water content calculation)  Reduce neurological deficit (mNSS)  Facilitate the production of new neurons (BrdU administration) | BDNF/TrKB path | Upregulated |
| Zhang et al.  (2020a) | GSRd | MCAO/R | SD rats | GSRd (10mg/kg) for 1d | NA | NA | NR2b/NMDAR | Downregulated |
| Zhou et al.  (2021a) | Storax | MCAO/R | Wistar rats | Storax (200/400mg/kg) for 1/28d | NA | Reduce neurological deficit (mNSS)  Increase angiogenesis and suppresses inflammation (immunohistochemical) | NF-κB | Downregulated |
| Zhao et al.  (2021) | BA | MCAO/R | SD rats | BA (50mg/kg) for 7d | NA | Reduce infarct size (TTC)  Inhibit neuronal autophagy (immunofluorescence) | SIRT1/FOXO1 | Upregulated |
| Hou et al.  (2010) | Silymarin | MCAO/R | Long-Evans rats | Silymarin (1/5/10ug/kg) for 1d | NA | Inhibit inflammatory response (immunohistochemical) | NF-κB  STAT-1 | Downregulated |
| Wang et al.  (2007) | Emodin-8-O-beta-D-glucoside | MCAO/R | Wistar rats | Emodin-8-O-beta-D-glucoside (2.5/5/10mg/kg) for 1d | vitamin C(20mg/kg) | Reduce neurological deficit (Longa)  Reduce infarct size (TTC)  Inhibit oxidative stress response (MDA assay) | NA |  |
| Leung et al.  (2020) | Emodin | MCAO/R | SD rats | Emodin (15mg/kg) for 1d | NA | Reduce infarct size (TTC) | GLT-1 | Upregulated |
| Xie et al.  (2023) | L-borneol | MCAO/R | SD rats | L-borneol (50/100/200mg/kg) for 3d | nimodipine(12mg/kg) | Reduce neurological deficit (mNSS)  Inhibit inflammatory response (immunohistochemical)  Reduce infarct size (TTC)  Improve regional cerebral blood flow disturbance (laser-Doppler) | p38 MAPK path | Upregulated |
| Ma et al.  (2023) | L-borneol/d-borneol | MCAO/R | SD rats | L-borneol/d-borneol (50/100/200mg/kg) for 14d | nimodipine(50/100/200mg/kg) for 14d | Reduce neurological deficit (mNSS)  Improve regional cerebral blood flow disturbance (laser-Doppler)  Promote astrocyte differentiation (immunofluorescence) | Wnt/β-catenin  Notch-1 | Downregulated |
| Huang et al.  (2022) | borneol | MCAO/R | C57BL/6 mice、Trpm8^-/-^ mice | Borneol (8%/16%/200mg/kg) for 6d | NA | Reduce infarct size (TTC)  Inhibit oxidative stress response (immunohistochemical)  Promote astrocyte and microglial proliferation (immunofluorescence)  Inhibit inflammatory response (Westernblot) | TRPM8 | Upregulated |
| Wang et al.  (2023a) | Scutellarin | MCAO/R | SD rats | Scutellarin (6/12mg/kg) | NA | Reduce infarct size (TTC) | Excitatory amino acid | Downregulated |
| Seo et al.  (2023) | SB extracts | MCAO/R | ICR mice | SB extracts (200mg/kg) for 3d | NA | Reduce neurological deficit (mNSS)  Reduce infarct size (TTC)  Inhibit inflammatory response (Westernblot) | RIPK1/3  NLRP3 | Downregulated |
| Yang et al.  (2020) | Procyanidins | MCAO/R | SD rats | Procyanidins (20/40/80mg/kg) for 1h | NA | Reduce neurological deficit (Longa)  Reduce infarct size (TTC)  Reduce cerebral edema (water content calculation) | TLR4/p38/NF-κB/NLRP3 | Downregulated |
| Yu et al.  (2019) | EA | MCAO/R | ICR mice | EA (10/25/50mg/kg) for 6h | NA | Reduce neurological deficit (mNSS)  Reduce infarct size (TTC)  Inhibit inflammatory response (Westernblot) | JNK path | Downregulated |
| Chen et al.  (2020) | Glycyrrhizin | MCAO/R | SD rats | Glycyrrhizin (15/30/60mg/kg) | rt-PA(10mg/kg) | Reduce cerebral edema (water content calculation)  Protect the blood-brain barrier (EB test) | ONOO-/HMGB1/TLR2/MMP9 | Downregulated |
| Wang et al.  (2019) | EK100 | MCAO/R | ICR mice | EK100 (30/60/120mg/kg) | rt-PA(10mg/kg) | Reduce infarct size (TTC)  Inhibit inflammatory response (immunohistochemical) | PI3K/Akt/GSK-3/β-catenin | Upregulated |
| Li et al.  (2021c) | ASIV | MCAO/R | C57BL/6 mice、Trpm8^-/-^ mice | ASIV (20/40mg/kg) | NA | Reduce neurological deficit (neurological deficit scores)  Inhibit brain infiltration by NK cells (Flow Cytometry)  Inhibit inflammatory response (Westernblot) | STAT3/CCL2 | Downregulated |
| Li et al.  (2013) | ASIV | MCAO/R | SD rats | ASIV (10/20mg/kg) | NA | Reduce neurological deficit (Bederson)  Reduce cerebral edema (water content calculation)  Protect the blood-brain barrier (EB test) | MMP-9  AQP4 | Downregulated |
| Shi et al.  (2021) | ASIV | MCAO/R | SD rats | ASIV (20mg/kg) for 10d | NA | Reduce neurological deficit (neurological deficit scores)  Reduce infarct size (TTC) | SIRT1/MAPT | Upregulated |
| Li et al.  (2021b) | ASIV | MCAO/R | SD rats | ASIV (40mg/kg) for 14d | NA | Reduce neurological deficit (mNSS)  Promote M2 microglia/macrophage polarization (immunofluorescence)  Promote neurogenesis and angiogenesis (BrdU administration)  Promote neurotrophic factor expression (Westernblot) | PPARγ | Upregulated |
| Mao et al.  (2017) | Gas-d | MCAO/R | SD rats | Gas-d (100mg/kg) | NA | Inhibit oxidative stress response (immunohistochemical) | Prx1/2/4/TLR4 | Downregulated |
| Lee et al.  (2012b) | DSE | MCAO/R | SD rats | DSE (30/100/300mg/kg) for 7d | NA | Reduce cerebral edema (water content calculation)  Protect the blood-brain barrier (EB test) | AQP4 | Downregulated |
| Bai et al.  (2024) | PQS | MCAO/R | SD rats | PQS (100/200mg/kg) | NA | Reduce neurological deficit (Bederson)  Reduce infarct size (TTC) | NA |  |
| Zhang et al.  (2024) | VOEX | MCAO/R | SD rats | VOEX (200/400/800mg/kg) | EGB761(100mg/kg) | Reduce neurological deficit (mNSS)  Reduce infarct size (TTC) | IL17A | Downregulated |
| Zhang et al.  (2023c) | Rus | MCAO/R | C57BL/6 mice | Rus (10mg/kg) for 3d at 17:00—19:00 | NA | Reduce neurological deficit (neurological deficit scores)  Reduce infarct size (TTC)  Inhibit oxidative stress response (MDA assay)  Inhibit inflammatory response (Westernblot) | Nrf2/HO-1 | Upregulated |
| Wang et al.  (2021) | TFCJ | MCAO/R | SD rats | TFCJ (10/20/40 mg/kg) for 7d | Xuesaitong(40mg/kg) | Reduce neurological deficit (neurological deficit scores)  Inhibit oxidative stress response (MDA assay)  Reduce infarct size (TTC) | PI3K/Akt/mTOR | Upregulated |
| Huai et al.  (2013) | L-NBP | MCAO/R | C57BL/6 mice | L-NBP (30mg/kg) for 7/28d | NA | Improve cognitive impairment (Morris Water Maze Experiment) | Akt | Upregulated |
| Wang et al.  (2020) | DL-NBP | MCAO/R | C57BL/7 mice | DL-NBP (6.5mg/kg) for 14d | NA | Reduce neurological deficit (Longa)  Protect the blood-brain barrier (EB test)  Promote angiogenesis (immunofluorescence) | HIF-1α/VEGF/Notch/Dll4 | Upregulated |
| Zhang et al.  (2014) | GL | MCAO/R | Wistar rats | GL (20mg/kg) for 3/7d | NA | Improve cognitive impairment (Platform Jumping Experiment)  Inhibit oxidative stress response (immunohistochemical)  Inhibit inflammatory response (immunohistochemical) | NA |  |
| Qi et al.  (2014) | HSYA | MCAO/R | SD rats | HSYA (2mg/kg) for 3d | NA | Reduce neurological deficit (neurological deficit scores)  Reduce infarct size (TTC)  Promote autophagy in penumbra neurons (immunohistochemistry) | Akt | Upregulated |
| Lee et al.  (2012a) | ES | MCAO/R | Wistar rats | ES (3/30/300mg/kg) | NA | Improve cognitive impairment (Y Maze)  Inhibit astrocyte and microglial activation (immunohistochemical) | COX-2 | Downregulated |
| Choi et al.  (2022) | GRex | MCAO/R | C57BL/7mice | GRex (31.25/62.5/125/250/500/1000mg/kg) | NA | Reduce neurological deficit (neurological deficit scores)  Reduce infarct size (TTC)  Inhibit inflammatory response (immunofluorescence) | NA |  |
| Teixeira et al.  (2023) | EO | MCAO/R | Wistar rats | EO (200mg/kg) for 14d | NA | Reduce neurological deficit (neurological deficit scores)  Alleviate ischemic injury of penumbra cells (immunohistochemical) | NA |  |
| Lin et al.  (2013) | Hyperforin | MCAO/R | SD rats | Hyperforin | NA | Reduce neurological deficit (neurological deficit scores)  Reduce infarct size (TTC)  Inhibit apoptosis (TUNEL staining) | TRPC6 | Downregulated |
| Jiang et al.  (2018b) | Vitexin | MCAO/R | SD rats | Vitexin (2mg/kg) | NA | Reduce infarct size (TTC)  Inhibit oxidative stress response (ELISA)  Inhibit inflammatory response (Westernblot)  Inhibit autophagy (immunohistochemical) | mTOR/Ulk1 | Upregulated |
| Chen et al.  (2014) | Honokiol | 2VO | SD rats | Honokiol (10/30mg/kg) | NA | Reduce infarct size (TTC)  Inhibit oxidative stress response (immunohistochemical)  Inhibit inflammatory response (Westernblot) | p38 MAPK  CHOP  nitrotyrosine | Downregulated |
| Wang et al.  (2023b) | ASIV | MCAO/R | SD rats | ASIV(28mg/kg) | NA | Improve related neurological dysfunction (balance beam score)  Reduce neurological deficit (Longa)  Reduce infarct size (TTC) | Nrf2 | Upregulated |
| Li et al.  (2024) | Sophoricoside | MCAO/R | C57BL/7mice | Sophoricoside(45/90mg/kg) | NA | Reduce neurological deficit (Berderson)  Reduce infarct size (TTC)  Inhibit apoptosis (TUNEL fluorescence staining)  Inhibit inflammatory response (immunofluorescence) | Bax/Bcl-2 | Downregulated |
| Yu et al.  (2022) | Formononetin | MCAO/R | SD rats | Formononetin(30mg/kg) for 3d | NA | Reduce neurological deficit (Longa)  Reduce infarct size (TTC) | JAK2/STAT3 | Downregulated |
| Zheng et al.  (2022) | Daidzein | MCAO/R | ICR mice | Daidzein(10/20/30mg/kg) for 14d | Edaravone(10mg/kg) for 14d | Reduce neurological deficit (Longa)  Reduce infarct size (TTC) | Akt/mTOR/BDNF | Downregulated |
| Zhao et al.  (2015) | Matrine | MCAO/R | ICR mice | Matrine(7.5/15/30mg/kg) for 7d | nimodipine (1 mg/kg) for 7d | Reduce neurological deficit (neurological deficit scores)  Reduce infarct size (TTC)  Inhibit apoptosis (Westernblot, RT-qPCR) | Bax/Bcl-2/Caspase-3 path | Downregulated |
| Pengyue et al.  (2017) | Breviscapine | MCAO/R | SD rats | Breviscapine(0.33mg/kg) for 7d | NA | Reduce neurological deficit (neurological deficit scores)  Reduce infarct size (TTC)  Reduce cerebral edema (calculated water content)  Inhibit autophagy (immunofluorescence) | NA |  |
| Cao et al.  (2011) | Scutellarin | MCAO/R | Mongolian gerbils | Scutellarin(50/100/200mg/kg) for 7d | NA | Inhibit apoptosis (Westernblot) | Caspase-3 path | Downregulated |
| Wang et al.  (2010) | Shikonin | MCAO/R | Kunming mice | Shikonin(12.5/25/50mg/kg) for 24h | Edaravone | Reduce neurological deficit (Longa)  Reduce infarct size (TTC)  Inhibit peroxidation (ROS assay) | NA |  |
| Guang et al.  (2013) | Rus | MCAO/R | C57BL/7mice | Rus(2.5/5/10mg/kg) | NA | Reduce neurological deficit (neurological deficit scores)  Reduce infarct size (TTC) | NF-κB | Downregulated |
| Xiong et al.  (2016) | ICA | MCAO/R | SD rats | ICA(10/30mg/kg) for 3d | NA | Reduce neurological deficit (Berderson)  Reduce infarct size (TTC) | PPARα  PPAR γ | Upregulated |
| Cheng et al.  (2021b) | ASD extract | 4VO | SD rats | ASD extract(250/500/1000mg/kg) for 24d | NA | NA | p38MAPK path | Upregulated |
| Liang et al.  (2014) | Formononetin | MCAO/R | SD rats | Formononetin(12.5/25/50mg/kg) for 14d | NA | Reduce neurological deficit (neurological deficit scores)  Reduce infarct size (TTC)  Reduce cerebral edema (water content calculation) | Bax/Bcl-2  PI3K/Akt | Upregulated(PI3K/Akt)  Downregulated(Bax/Bcl-2) |
| Park et al.  (2009) | ESF | MCAO/R | SD rats | ESF(20mg/kg) | carnosine(50mg/kg) | Reduce infarct size (TTC) | NA |  |
| Mahmood et al.  (2017) | SAA | MCAO/R | C57BL/7mice | SAA(1/5mg/kg) for 7d | NA | Reduce neurological deficit (neurological deficit scores)  Reduce infarct size (TTC) | ONOO- | Downregulated |
| Lin et al.  (2015) | Methylophiopogonanone A | MCAO/R | SD rats | Methylophiopogonanone A(1.25/2.5/5mg/kg) for 7d | NA | Reduce neurological deficit (neurological deficit scores)  Reduce infarct size (TTC)  Reduce cerebral edema (calculated water content) | MMP-9 | Downregulated |
| Dai et al.  (2018) | Scutellarin | MCAO/R | SD rats | Scutellarin(100mg/kg) for 12d | valproic acid(300mg/kg) for 72h | Reduce neurological deficit (neurological deficit scores)  Reduce infarct size (TTC) | NA |  |
| Chen et al.  (2019) | ASIV | MCAO/R | SD rats | ASIV(2ug/kg) for 7d | NA | Improvement of related neurological dysfunction (Water Maze Test, Motor Test)  Reduce infarct size (TTC) | NA |  |
| Hui et al.  (2017) | PNS | MCAO/R | SD rats | PNS(50mg/kg) for 14d | cyclopamine(10mg/kg) for 7d | Reduce neurological deficit (Longa)  Reduce infarct size (TTC)  Improve regional cerebral blood flow disturbance (Micro-PET/CT) | SHH | Upregulated |
