# Supplementary material for: Natural herbal extract roles and mechanisms in treating cerebral ischemia: A systematic review
Source: Front Pharmacol. 2024 Aug 2;15:1424146. doi: 10.3389/fphar.2024.1424146 (PMC11327066; doi:10.3389/fphar.2024.1424146)
Supplement: Supplementary file 2 [file Table2.docx]

**Supplementary Table 2. Extraction Methods and Toxicity of NHEs**

| Author(Years) | Extracts | Source | Voucher number (storage mechanism) | Extracting part | Extraction scheme | Extraction solvent | Toxicity/side effect |
| --- | --- | --- | --- | --- | --- | --- | --- |
| Li et al.  (2021a) | LHA | Leonurus japonicus Houtt. | No.20170809(group’s laboratory) | NA | NA | NA | NA |
| Zhang et al.  (2017a) | LHA | Leonurus japonicus Houtt. | NA | NA | NA | NA | NA |
| Rodrigues et al.  (2017) | EEEV | Erythrina velutina Willd. | No.44802 (Herbarium Prisco Bezerra, Department of Biology, Federal University of Ceará-Brazil) | stem bark | The extract was obtained from previously dried vegetable drugs(80±5°C) in an oven with renewal and air circulation for 24 h and pulverized in a slicer with adequate granulometry, which was subjected to extraction by percolation preceded by maceration (24) in the ratio 1:2 (w/w) of vegetable and drug solvent. The used organic solvent(ethanol) evaporated at the end of the extraction process in a rotary evaporator at 60 °C. | ethanol | NA |
| Wang et al.  (2022) | catalpol | Rehmannia glutinosa | NA | NA | NA | NA | The results of the hemolysis experiment showed that no hemolysis was observed, although the concentration of catalpol was different. The results of cilia toxicity experiments showed that catalpol had no obvious effect on the movement of intranasal cilia. |
| Cheng et al.  (2021a) | YZR extracts | Alpinia oxyphylla Miq. | NA | NA | NA | NA | NA |
| Zhao et al.  (2017) | MO | Melilotus officinalis | NA | ALL | Dried plant was chopped into small pieces and powered coarsely. Pieces of plants were defatted by keeping it with petroleum ether for the duration of 72 h and thereafter powder was dried. Aquesous extract of this defatted powder was obtained by the process of maceration | Water | NA |
| Zhang et al.  (2018) | DGMI | Ginkgo biloba L. | NA | NA | NA | NA | NA |
| Saleem et al.  (2008) | EGB761 | Ginkgo biloba L. | NA | NA | NA | NA | NA |
| Yang et al.  (2018) | EGB761  GB | Ginkgo biloba L. | NA | NA | NA | NA | NA |
| Li et al.  (2007) | GB | Ginkgo | NA | NA | NA | NA | NA |
| Fei et al.  (2017) | SCED | Salvia miltiorrhiza Bunge | DS150401 (Herbarium of Department of Pharmaceutical analysis, China Pharmaceutical University, Jiangsu, China) | Root | 300g of dried roots of Salvia miltiorrhiza were cleaned, dried in the shade, and ground into fine coarse powder and then extracted with the supercritical CO2 extraction instrument at 60 °C under 30 MPa for 2 h at a flow of 20 L/h. Ethanol (95%) was used as a co-solvent at a flow of 0.8 g/min. Crude extract was obtained after the evaporation of ethanol in rotary evaporator. | 95% ethanol | NA |
| Yu et al.  (2016) | OLE | Canarium oleosum | NA | NA | NA | NA | We performed measurements of heart rate, temperature, pH, pO2, pCO2 and glucose levels in mice 30 min before ischemia, 75 min after ischemia and 30 min after reperfusion, respectively. The results showed that OLE treatment had no significant effect on the physiological parameters of mice. |
| Liu et al.  (2022) | NGR1 | Panax notoginseng | NA | NA | NA | NA | We applied CCK-8 cell experiments in vitro, and the results showed that 5-200 μM NGR1 had no obvious toxicity to OGD-treated cells. |
| Gao et al.  (2022) | PNS | Panax notoginseng | NA | NA | NA | NA | NA |
| Liu et al.  (2021a) | NGR1 | Panax notoginseng | NA | NA | NA | NA | The results of CCK-8 cell viability assay showed that when the concentration of the two drugs reached 200 μM, there was no damage and inhibition to the cells. It is suggested that NGR1 and NBP are relatively safe and have no obvious cytotoxicity. |
| Tu et al.  (2018) | NGR1 | Panax notoginseng | NA | NA | NA | NA | NA |
| Wang et al.  (2017a) | GSRb1 | Panax notoginseng | NA | NA | NA | NA | NA |
| Ling et al.  (2021) | SAA | Salvia miltiorrhiza Bunge | no.17110723 (Chiatai qingchunbao Pharmaceutical Co., Ltd.) | ALL | 100g of dried Salvia miltiorrhiza pieces was weighed and dissolved in 50 ml water followed by reflux extraction with a gradient ethanol aqueous solution of 10% to 95%, filter and remove solid impurities, in which the temperature of immersion is about 50 degree centigrade. | ethanol | The toxicity of SAA to microglia was detected by MTT assay, and 62.5, 125 and 250 μg/ml SAA were selected as safe doses. |
| Chien et al.  (2016) | SAA | Salvia miltiorrhiza Bunge | NA | NA | NA | NA | NA |
| Jiang et al.  (2011) | SAA | Salvia miltiorrhiza Bunge | NA | NA | NA | NA | NA |
| Song et al.  (2019) | SAA | Salvia miltiorrhiza Bunge | NA | NA | NA | NA | NA |
| Liu et al.  (2021b) | SAA | Salvia miltiorrhiza Bunge | NA | NA | NA | NA | SAA had no effect on animal weight, indicating that the drug itself had no adverse effect on animal weight. |
| Luan et al.  (2020) | SA | Salvia miltiorrhiza Bunge | NA | NA | NA | NA | NA |
| Zhang et al.  (2017b) | SA | Salvia miltiorrhiza Bunge | NA | NA | NA | NA | NA |
| Liu et al.  (2010) | TSA | Salvia miltiorrhiza Bunge | NA | NA | NA | NA | NA |
| Yang et al.  (2016) | GSRd | Panax ginseng | NA | NA | NA | NA | NA |
| Zhang et al.  (2019) | GSF1 | Panax ginseng | NA | NA | NA | NA | NA |
| Zhou et al.  (2014) | GSRg1 | Panax ginseng | NA | NA | NA | NA | NA |
| Yuan et al.  (2020) | PF11 | Panax ginseng | NA | NA | NA | NA | NA |
| Zhang et al.  (2020a) | GSRd | Panax ginseng | NA | NA | NA | NA | NA |
| Zhou et al.  (2021a) | Storax | Liquidambar orientalis | No.120113180803077LY (Specimen Museum of traditional Chinese Medicine, Tianjin University of Traditional Chinese Medicine) | wounded bark | Storax was added to tween-80 (Sigma-Aldrich, P8074，BioXtra) drop_x0002_wise with continuous grinding for thoroughly emulsifying, and diluted with pure water to needed concentration. | tween-80 | Initially, our experiments showed that rats pretreated with 0.8 g/kg Storax for 5 days developed a mild toxic reaction, manifested by slow weight gain. The results showed that 0.4 g/kg Storax had a better anti-inflammatory effect than 0.2 g/kg. Therefore, we chose a dose of 0.4 g/kg for long-term observation, which is 4 times higher than the dose used in humans. The results of the study validate our hypothesis and suggest that 0.4 g/kg may be the best dose for preclinical evaluation. |
| Zhao et al.  (2021) | BA | Betula | NA | NA | NA | NA | NA |
| Hou et al.  (2010) | Silymarin | Silybum marianum | NA | NA | NA | NA | Blood gas data (pH, pCO2, pO2) and physiological data (blood pressure, heart rate) were monitored without significant differences between groups. |
| Wang et al.  (2007) | Emodin-8-O-beta-D-glucoside | Rheum palmatum L. | NA | NA | NA | NA | NA |
| Leung et al.  (2020) | Emodin | Rheum palmatum L. | NA | NA | NA | NA | We evaluated the toxicity of different doses of emodin and showed inhibition of PC12 cell activity by 10uM emodin. |
| Xie et al.  (2023) | L-borneol | Dipterocarpus turbinatus | NA | NA | NA | NA | NA |
| Ma et al.  (2023) | L-borneol/d-borneol | Dipterocarpus turbinatus | NA | NA | NA | NA | In this study, the effects of L-Borneolum and Borneolum on gastric mucosa of t-MCAO rats were studied. The results showed that L-Borneolum and Borneolum had no obvious damage to gastric mucosa, liver and kidney. |
| Ma et al.  (2021) | L-borneol | Dipterocarpus turbinatus | NA | NA | NA | NA | NA |
| Huang et al.  (2022) | borneol | Dipterocarpus turbinatus | NA | NA | NA | NA | NA |
| Wang et al.  (2023a) | Scutellarin | Scutellaria baicalensis Georgi | NA | NA | NA | NA | NA |
| Seo et al.  (2023) | SB extracts | Scutellaria baicalensis Georgi | 2009150001 (Department of Herbal Pharmacology, College of Korean Medicine, Gachon University) | Root | The dried root of Scutellaria baicalensis was extracted using a reflux device for 3 h at 85 ◦C and a 10:1 ratio of 30% ethanol. | 30% ethanol | NA |
| Zhang et al.  (2023b) | SHPL-49 | Rhodiola rosea L. | NA | NA | NA | NA | The cytotoxicity of SHPL-49 on PC-12 cells was evaluated by CCK-8 assay, and there was no effect on cell viability at concentrations below 400 μM. |
| Li et al.  (2012a) | Galangin | Alpinia officinarum Hance | NA | NA | NA | NA | NA |
| Yang et al.  (2020) | Procyanidins | Vitis vinifera L. | NA | NA | NA | NA | NA |
| Liu et al.  (2017) | KuA | Lycium barbarum L. | NA | NA | NA | NA | NA |
| Li et al.  (2015a) | T-VA | Ligusticum sinense | NA | NA | NA | NA | During the oral maximum tolerated dose of T-VA (5.4 g/kg/d), none of the male and female mice showed symptoms of toxicity or abnormal behavior for two weeks. There were no significant differences in body weight and food intake between T-VA and control groups at the end of the experiment. |
| Yu et al.  (2019) | EA | Albizzia julibrissin Durazz. | NA | NA | NA | NA | NA |
| Chen et al.  (2020) | Glycyrrhizin | Glycyrrhiza uralensis Fisch. | NA | NA | NA | NA | NA |
| Wang et al.  (2019) | EK100 | Antrodia camphorata | NA | NA | NA | NA | NA |
| Li et al.  (2021c) | ASIV | Astragalus membranaceus (Fisch.) Bunge | NA | NA | NA | NA | NA |
| Li et al.  (2013) | ASIV | Astragalus membranaceus (Fisch.) Bunge | NA | NA | NA | NA | NA |
| Shi et al.  (2021) | ASIV | Astragalus membranaceus (Fisch.) Bunge | NA | NA | NA | NA | NA |
| Li et al.  (2021b) | ASIV | Astragalus membranaceus (Fisch.) Bunge | NA | NA | NA | NA | NA |
| Liu et al.  (2019a) | KRGP | Korean red ginseng | NA | NA | NA | NA | NA |
| Jiang et al.  (2018a) | celastro | Tripterygium wilfordii Hook. f. | NA | NA | NA | NA | NA |
| Mao et al.  (2017) | Gas-d | Gastrodia elata BI. | NA | NA | NA | NA | The results of MTT assay showed that neither Gas nor Gas-D (1-10μM) had significant cytotoxicity. |
| Lee et al.  (2012b) | DSE | Sesamum indicum L. | 1978ME-100-4 (Rural Develop_x0002_ment Administration, Republic of Korea) | seed | Sesame seeds(500 g) were ground and defatted with n-hexane (1L×3)to obtain defatted sesame flour (250 g). The defatted flour was extracted with 1 L of 80% (vol/vol) MeOH to obtain the DSE (35g) | 80% MeOH | NA |
| Bai et al.  (2024) | PQS | Panax quinquefolius L. | NA | NA | NA | NA | NA |
| Zhang et al.  (2024) | VOEX | Verbena officinalis L. | NA | aboveground part | In brief, the aboveground part of VO was macerated in ethanol (95%, 8 l, sample/ethanol ratio, 1:8; w/w) for 1 h and then extracted by heating to reflux three times for 1h each time. The suspensions that had been filtered and mixed were gathered and concentrated. | 95% ethanol | The results of cytotoxic activity showed that verbenalin, hastatoside, acteoside and apigenin had no significant effect on cell viability in the concentration range of 0-200uM, while luteolin (0-25uM) and hispidulin (0-100uM) had no effect on cell viability. |
| Zhang et al.  (2023c) | Rus | Ophiopogon japonicus | NA | NA | NA | NA | NA |
| Wang et al.  (2021) | TFCJ | Chuju(Bellis perennis L.) | NA | ALL | Air-dried TFCJ was treated twice with 70% aqueous ethanol (1:25,w/v), mixed and sonicated 60◦C for 40 min, then concentrated in a rotary evaporato. | 70% ethanol | NA |
| Huai et al.  (2013) | L-NBP | Apium graveolens L. | NA | NA | NA | NA | NA |
| Wang et al.  (2020) | DL-NBP | Apium graveolens L. | NA | NA | NA | NA | NA |
| Ferreira et al.  (2023) | EDAC | Amburana cearensis | 13734 (Herbarium of the Biology Institute of the Federal University of Bahia) | seed | In brief, A.cearensis seeds were slow dried at 40°C for 24h and the EDAC extract was prepared by subjecting dried and fine-ground seeds to maceration in dichloromethane for 72h, after which the solvent was completely removed in a rotary evaporator. | dichloromethane | NA |
| Zhou et al.  (2021b) | PNS | Panax notoginseng | NA | NA | NA | NA | NA |
| Zhang et al.  (2014) | GL | Ganoderma lucidum | NA | NA | NA | NA | NA |
| Qi et al.  (2014) | HSYA | Carthamus tinctorius L. | NA | NA | NA | NA | NA |
| Sun et al.  (2015) | Asiaticoside | Centella asiatica | NA | NA | NA | NA | NA |
| Lee et al.  (2012a) | ES | Eleutherococcus senticosus | #HP060 (Department of Herbal Pharmacology, College of Oriental Medicine, Kyung Hee University) | stem bark | Eleutherococcus senticosus (150g) was extracted with 70% ethanol (3000 ml) for 3h at 80◦C in a reflux apparatus. The extract was filtrated and concentrated under reduced pressure, then, lyophilized to yield a dark brown powder. | 70% ethanol | NA |
| Choi et al.  (2022) | GRex | Glycyrrhiza uralensis Fisch. | No.19GR-2483 (Herbarium of School of Korean Medicine, Pusan National University) | ALL | To prepare GRex, 200 g of GR was immersed in 2000 mL of methanol at 25 ◦C for three days, then filtered using filter paper, and the supernatant was separated. Then, 1000 mL of methanol was added to the filtered GR residue for two days, and the mixture was filtered again. The supernatant was collected twice and concentrated under reduced pressure and freeze-dried. | methanol | NA |
| Teixeira et al.  (2023) | EO | Euterpe oleracea Mart. | NA | NA | NA | NA | NA |
| Lin et al.  (2013) | Hyperforin | Hypericum perforatum L. | NA | NA | NA | NA | NA |
| Jiang et al.  (2018b) | Vitexin | Vitex negundo L. | NA | NA | NA | NA | NA |
| Qin et al.  (2012) | PAL extracts | Potentilla anserina L. | NA | NA | NA | NA | NA |
| Chen et al.  (2014) | Honokiol | Houpoea officinalis | NA | NA | NA | NA | NA |
| Wan et al.  (2022) | Triptolide | Tripterygium wilfordii Hook. f. | NA | NA | NA | NA | Chronic triptolide treatment at 20μg/kg/d has no hepatotoxic or nephrotoxic effects after chronic cerebral hypoperfusion. |
| Feng et al.  (2012) | LIG | Angelica sinensis | NA | NA | NA | NA | NA |
| Peng et al.  (2022a) | LIG | Angelica sinensis | NA | NA | NA | NA | NA |
| Yang et al.  (2022a) | SAA | Salvia miltiorrhiza Bunge | NA | NA | NA | NA | NA |
| Tan et al.  (2022) | Que | Fruits and vegetables | NA | NA | NA | NA | NA |
| Liu et al.  (2019b) | CZ-7 | Clausena lansium | NA | NA | NA | NA | Preliminary acute toxicological test results showed that CZ-7 oral dose of 1 g/kg had no toxic and side effects on ICR mice. |
| Zhang et al.  (2023a) | Honokiol | Houpoea officinalis | NA | NA | NA | NA | CCK-8 results showed that neither honokiol (5-50 μmol/L) nor magnolol (5-50 μmol/L) affected cell viability. |
| Chen et al.  (2018a) | HAR | Scrophularia ningpoensis Hemsl. | NA | NA | NA | NA | The results showed that harpagoside did not affect basal physiological functions and had no effect on metabolic parameters and general exercise capacity. |
| Lee et al.  (2015) | Fructus extracts | Prunus mume Siebold & Zucc. | NA | NA | NA | NA | Fructus extracts extract had no toxic effect in general behavior and changes in mortality. |
| Li et al.  (2012b) | Polydatin | Reynoutria japonica Houtt. | No.PC001 (Herbarium of the Pharmacy School of the Second military Medical University, Shanghai, China) | Root | The air-dried roots of Polygonum cuspidatum Sieb. et Zucc.(20kg) were extracted with 70% aqueous EtOH under reflux for 2 h(3 ×200l). EtOH was evaporated under vacuum and the hydropho_x0002_bic substances precipitated, which was filtered. | 70% ethanol | NA |
| Shi et al.  (2020) | Gas | Gastrodia elata BI. | NA | NA | NA | NA | NA |
| Wu et al.  (2023) | Gas | Gastrodia elata BI. | NA | NA | NA | NA | NA |
| Yao et al.  (2021) | EGB761 | Ginkgo biloba L. | NA | NA | NA | NA | NA |
| Kim et al.  (2016) | GBE | Ginkgo biloba L. | NA | NA | NA | NA | NA |
| Niu et al.  (2020) | EF | Epimedium brevicornu Maxim. | NA | NA | NA | NA | NA |
| Li et al.  (2015b) | ICA | Epimedium brevicornu Maxim. | NA | NA | NA | NA | NA |
| Wan et al.  (2017) | GSRd | Panax ginseng | NA | NA | NA | NA | NA |
| Zong et al.  (2019) | CK | Panax ginseng | NA | NA | NA | NA | NA |
| Zhu et al.  (2018) | GSRg1 | Panax ginseng | NA | NA | NA | NA | NA |
| Hwang et al.  (2011) | SB extracts | Scutellaria baicalensis Georgi | NA | Root | Briefly, Scutellaria baicalensis (50g)was extracted with 2 L of boiling water for 2 h, filtered, and then lyophilized. | Water | NA |
| Ahad et al.  (2023) | CTRF | Clitoria ternatea L. | no.11876 (School of Biological Sciences, Universiti Sains Malaysia) | Root | The air-dried powdered roots were then macerated successively with hexane, followed by chloroform and then methanol. The extraction was carried out twice using each solvent (3 days each), and the extract obtained from each extraction using the same solvent was combined and subsequently evaporated to dryness in vacuo at low temperature (<40ºC). | hexane/chloroform/methanol | NA |
| Damodaran et al.  (2018) | CTRF | Clitoria ternatea L. | 11551 (School of Biological Sciences) | Root | Dried and coarse powders of CTR (1 300 g) were macerated with 13L of absolute methanol at a ratio 1:10 for 72 hours with occasional shaking. The extract was filtered using filter paper (Whatman No 1). The residues were extracted with fresh solvent twice(for a total of 3 extractions), and the extracts were combined. The crude extract was then concentrated at a reduced pressure using a rotary evaporator. | methanol | NA |
| Tiang et al.  (2020) | XEFGM  α-MG | Garcinia mangostana L. | No.11247 (the herbarium, School of Biological Sciences, Universiti Sains Malaysia, Malaysia) | dried pericarp | The dried and powdered pericarp of G. mangostana was extracted with methanol by using maceration method for 3 days at 60°C at raw material to solvent ratio of 1 : 10 (w/v). Fresh solvents were replenished every day and the result_x0002_ing extracts were filtered through filter paper. The pooled extracts were evaporated under vacuum and lyophilized. | methanol | Mice were assessed for acute toxicity of α-MG, reporting that at single doses up to 1000 mg/kg, no signs of toxicity and death were observed for 14 days during the study period. |
| Hosseinzadeh et al.  (2012) | CSL extracts  crocin | Crocus sativus L. | NA | saffron stigma | Briefly, saffron stigma powder(10g) was suspended in 25mL ethanol 80% at 0ºC and shaken by vortex for 2min. After centrifugation at 4000rpm for 10min, the supernatant was separated.Then 25mL of 80% ethanol was added to the sediment and the extraction was repeated again. This step was repeated six more times. | 80% ethanol | NA |
| Kim et al.  (2023) | AA | Artemisia annua L. | NA | ALL | Dried AA (250 g, Bukseorak Co. Ltd., Gangwon-do, Republic of Korea) was extracted in 2000 ml water at 50 ◦C for 48 h and then concentrated under vacuum using a rotatory evaporator to obtain an extract with a yield of 32% and concentration of 199.9 mg/ml. | Water | NA |
| Guang et al.  (2006) | pinocembrin | propolis | NA | NA | NA | NA | NA |
| Wang et al.  (2023b) | ASIV | Astragalus membranaceus (Fisch.) Bunge | NA | NA | NA | NA | NA |
| Li et al.  (2024) | Sophoricoside | Sophora japonica L. | NA | NA | NA | NA | We injected sophoricoside 90 mg/kg intraperitoneally into mice. 24 hours later, the liver and kidney were taken for HE staining. The results showed no significant damage to the liver and kidneys compared to the DMSO-injected group. |
| Yu et al.  (2022) | Formononetin | Trifolium pratense L. | NA | NA | NA | NA | NA |
| Zheng et al.  (2022) | Daidzein | Pueraria lobata (Willd.) Ohwi | PLP-20–0628 (Central Laboratory, Changchun Normal University) | Root | P. lobate (100 g each) received the crushing and 2 h extraction 3 times with reflux inside 70% ethanol under 1,000 ml. | 70% ethanol | NA |
| Zhao et al.  (2015) | Matrine | Sophora flavescens Aiton | NA | NA | NA | NA | NA |
| Pengyue et al.  (2017) | Breviscapine | Erigeron breviscapus (Vaniot) Hand.-Mazz. | NA | NA | NA | NA | NA |
| Cao et al.  (2011) | Scutellarin | Scutellaria baicalensis Georgi | NA | NA | NA | NA | NA |
| Wang et al.  (2010) | Shikonin | Lithospermum erythrorhizon Siebold & Zucc. | NA | NA | NA | NA | NA |
| Guang et al.  (2013) | Rus | Ophiopogon japonicus (Thunb.) Ker Gawl. | NA | NA | NA | NA | The physiological parameters of mice were monitored before and after ischemia induction and after Ruscogenin induction. The results showed that the differences in body weight, body temperature, pO2, pCO2, pH and other aspects between the different experimental groups did not reach statistical significance. There were no significant differences in changes in physiological parameters between groups at the beginning and end of the experimental protocol. In addition, Ruscogenin had no significant effect on cerebrovascular blood flow when measured before and after middle cerebral artery occlusion (MCAO). |
| Zhang et al.  (2013) | Luteolin | Ixeris sonchifolia (Maxim.) Hance | NA | aerial part | The aerial part of I. sonchifolia Hance (5 kg) was cut into small pieces (3 cm) and extracted three times with 95% ethanol at 60 _x0003_ C for 3 h. | 95% ethanol | NA |
| Xiong et al.  (2016) | ICA | Epimedium brevicornu Maxim. | NA | NA | NA | NA | NA |
| Cheng et al.  (2021b) | ASD extract | Angelica sinensis (Oliv.) Diels | A1306801(CSZ Ligang Laboratory) | Root | In brief, the dried roots of ASD were extracted using boiling water for 1.5 h and then the aqueous extract and essential oils from ASD were collected. | water | NA |
| Liang et al.  (2014) | Formononetin | Trifolium pratense L. | NA | NA | NA | NA | NA |
| Park et al.  (2009) | ESF | Sophora flavescens Aiton | A0093 (Depart_x0002_ment of Botany, Natural Products Research Institute of Seoul National university, Republic of Korea) | Root | Air-dried roots of S. flavescens were chopped into small pieces and extracted three times with MeOH at room temperature. The MeOH extract was evaporated to dryness under reduced pressure and then successively partitioned between H2O and hexane,and then EtOAc. | MeOH | ESF was not cytotoxic at the highest dose (20 mg/ml). |
| Mahmood et al.  (2017) | SAA | Salvia miltiorrhiza Bunge | NA | Root | The material was extracted with water un_x0002_der reflux three times each for at least 2h. | Water | NA |
| Lin et al.  (2015) | Methylophiopogonanone A | Ophiopogon japonicus (Thunb.) Ker Gawl. | NA | NA | NA | NA | NA |
| Dai et al.  (2018) | Scutellarin | Scutellaria baicalensis Georgi | NA | NA | NA | NA | NA |
| Chen et al.  (2019) | ASIV | Astragalus membranaceus (Fisch.) Bunge | NA | NA | NA | NA | NA |
| Hui et al.  (2017) | PNS | Panax notoginseng | NA | NA | NA | NA | NA |
