# Supplementary material for: Natural herbal extract roles and mechanisms in treating cerebral ischemia: A systematic review
Source: Front Pharmacol. 2024 Aug 2;15:1424146. doi: 10.3389/fphar.2024.1424146 (PMC11327066; doi:10.3389/fphar.2024.1424146)
Supplement: Supplementary file 3 [file Table3.docx]

**Supplementary Table 3. Bias risk assessments for selected studies.** A; Passed peer review. B; Followed randomness principles. C; Blinding was adopted. D; Sample size calculated. E; Complied with animal welfare regulations. F; Declared a conflict of interest

| No. | A | B | C | D | E | F |
| --- | --- | --- | --- | --- | --- | --- |
| 1 | √ | √ | × | × | √ | √ |
| 2 | √ | √ | × | × | √ | √ |
| 3 | √ | √ | × | × | √ | × |
| 4 | √ | √ | √ | × | √ | √ |
| 5 | √ | √ | × | × | √ | √ |
| 6 | √ | × | × | × | √ | √ |
| 7 | √ | √ | √ | × | √ | × |
| 8 | √ | × | × | × | √ | √ |
| 9 | √ | × | × | × | √ | × |
| 10 | √ | × | × | × | √ | × |
| 11 | √ | √ | √ | × | √ | √ |
| 12 | √ | √ | × | × | √ | × |
| 13 | √ | √ | √ | × | √ | √ |
| 14 | √ | √ | × | × | √ | √ |
| 15 | √ | √ | × | × | √ | √ |
| 16 | √ | √ | × | × | √ | × |
| 17 | √ | √ | × | × | √ | √ |
| 18 | √ | √ | × | × | √ | √ |
| 19 | √ | √ | × | × | √ | √ |
| 20 | √ | √ | × | × | √ | × |
| 21 | √ | √ | × | × | √ | × |
| 22 | √ | √ | √ | × | √ | √ |
| 23 | √ | × | × | × | √ | √ |
| 24 | √ | √ | × | × | √ | √ |
| 25 | √ | √ | √ | × | √ | √ |
| 26 | √ | √ | × | × | √ | √ |
| 27 | √ | √ | × | × | √ | √ |
| 28 | √ | √ | √ | × | √ | √ |
| 29 | √ | √ | √ | × | √ | √ |
| 30 | √ | × | × | × | √ | √ |
| 31 | √ | √ | × | × | √ | √ |
| 32 | √ | √ | × | × | √ | √ |
| 33 | √ | × | √ | × | √ | × |
| 34 | √ | √ | × | × | √ | × |
| 35 | √ | × | × | × | √ | × |
| 36 | √ | √ | × | × | √ | √ |
| 37 | √ | √ | √ | × | √ | √ |
| 38 | √ | √ | √ | × | √ | × |
| 39 | √ | √ | √ | × | √ | √ |
| 40 | √ | √ | × | × | √ | √ |
| 41 | √ | × | × | × | √ | √ |
| 42 | √ | √ | × | × | √ | √ |
| 43 | √ | √ | × | × | √ | × |
| 44 | √ | √ | × | × | √ | √ |
| 45 | √ | √ | √ | × | √ | √ |
| 46 | √ | √ | × | × | √ | √ |
| 47 | √ | √ | √ | × | √ | √ |
| 48 | √ | √ | √ | √ | √ | √ |
| 49 | √ | √ | × | × | √ | √ |
| 50 | √ | √ | √ | × | √ | √ |
| 51 | √ | √ | √ | × | √ | √ |
| 52 | √ | √ | √ | × | √ | × |
| 53 | √ | √ | √ | × | √ | √ |
| 54 | √ | √ | √ | × | √ | √ |
| 55 | √ | √ | √ | × | √ | √ |
| 56 | √ | √ | √ | × | √ | √ |
| 57 | √ | × | × | × | √ | √ |
| 58 | √ | √ | × | × | √ | √ |
| 59 | √ | √ | √ | × | √ | √ |
| 60 | √ | √ | √ | × | √ | √ |
| 61 | √ | √ | √ | × | √ | × |
| 62 | √ | √ | √ | × | √ | × |
| 63 | √ | × | × | × | √ | × |
| 64 | √ | √ | × | × | √ | √ |
| 65 | √ | √ | × | × | √ | √ |
| 66 | √ | √ | × | × | √ | √ |
| 67 | √ | √ | √ | × | √ | √ |
| 68 | √ | √ | × | × | √ | × |
| 69 | √ | √ | × | × | √ | × |
| 70 | √ | × | × | × | √ | √ |
| 71 | √ | √ | √ | × | √ | √ |
| 72 | √ | √ | √ | × | √ | √ |
| 73 | √ | √ | × | × | √ | √ |
| 74 | √ | √ | × | × | √ | √ |
| 75 | √ | √ | × | × | √ | √ |
| 76 | √ | √ | × | × | √ | √ |
| 77 | √ | √ | × | × | √ | × |
| 78 | √ | √ | × | × | √ | √ |
| 79 | √ | √ | × | × | √ | √ |
| 80 | √ | √ | × | × | √ | √ |
| 81 | √ | √ | × | × | √ | √ |
| 82 | √ | √ | × | × | √ | √ |
| 83 | √ | √ | × | × | √ | × |
| 84 | √ | × | × | × | √ | √ |
| 85 | √ | × | × | × | √ | × |
| 86 | √ | √ | × | × | √ | √ |
| 87 | √ | √ | × | × | √ | √ |
| 88 | √ | √ | × | × | √ | √ |
| 89 | √ | × | × | × | √ | √ |
| 90 | √ | √ | × | × | √ | √ |
| 91 | √ | √ | × | × | √ | × |
| 92 | √ | × | × | × | √ | √ |
| 93 | √ | √ | × | × | √ | √ |
| 94 | √ | √ | √ | × | √ | √ |
| 95 | √ | × | × | × | √ | × |
| 96 | √ | √ | × | × | √ | √ |
| 97 | √ | √ | × | × | √ | √ |
| 98 | √ | √ | × | × | √ | √ |
| 99 | √ | × | × | × | √ | √ |
| 100 | √ | √ | × | × | √ | √ |
| 101 | √ | √ | × | × | √ | × |
| 102 | √ | √ | √ | × | √ | √ |
| 103 | √ | √ | × | × | √ | √ |
| 104 | √ | √ | × | × | √ | √ |
| 105 | √ | √ | √ | × | √ | × |
| 106 | √ | √ | √ | × | √ | × |
| 107 | √ | √ | × | × | √ | √ |
| 108 | √ | √ | × | × | √ | × |
| 109 | √ | √ | √ | × | √ | × |
| 110 | √ | √ | × | × | √ | × |
| 111 | √ | √ | × | × | √ | √ |
| 112 | √ | √ | √ | × | √ | √ |
| 113 | √ | √ | × | × | √ | √ |
| 114 | √ | √ | × | × | √ | √ |
| 115 | √ | √ | × | × | √ | × |
| 116 | √ | × | × | × | √ | √ |
| 117 | √ | √ | √ | × | √ | × |
| 118 | √ | √ | √ | × | √ | √ |
| 119 | √ | √ | √ | × | √ | √ |
| 120 | √ | √ | √ | × | √ | √ |
